# Circulating immune landscape in melanoma patients undergoing anti-PD1 therapy reveals key immune features according to clinical response to treatment

**DOI:** 10.3389/fimmu.2024.1507938

**Published:** 2024-12-02

**Authors:** Eleonora Sosa Cuevas, Stéphane Mouret, Guillaume Vayssière, Siham Kerboua, Pauline Girard, Jean-Paul Molens, Marc Manceau, Julie Charles, Philippe Saas, Caroline Aspord

**Affiliations:** ^1^ Institute for Advanced Biosciences, Team: Epigenetics, Immunity, Metabolism, Cell Signaling & Cancer, Inserm U 1209, CNRS UMR, Université Grenoble Alpes, Grenoble, France; ^2^ R&D Laboratory, Etablissement Français du Sang Auvergne-Rhône-Alpes, Grenoble, France; ^3^ Dermatology, Allergology & Photobiology Department, CHU Grenoble Alpes, Univ. Grenoble Alpes, Grenoble, France; ^4^ Department of Medicine, Clinical Investigation Center, CHU Grenoble Alpes, Univ. Grenoble Alpes, Grenoble, France

**Keywords:** immunomonitoring, anti-PD1 therapy, melanoma, clinical response, T cells

## Abstract

**Introduction:**

Immune checkpoint blockers (ICB) bring unprecedented clinical success, yet many patients endure immune mediated adverse effects and/or fail to respond. Predictive signatures of response to ICB and mechanisms of clinical efficacy or failure remain understudied. DC subsets, in network with conventional αβ T (T_conv_), NK, γδ T and iNKT cells, harbor pivotal roles in tumor control, yet their involvement in response to ICB remained underexplored.

**Methods:**

We performed an extensive longitudinal monitoring of circulating immune cells from melanoma patients treated with first-line anti-PD1, before (T0) and during treatment. We assessed the phenotypic and functional features of DC and effector cells’ subsets by multi-parametric flow cytometry and ProcartaPlex^®^ dosages.

**Results:**

We revealed differences according to response to treatment and modulations of patterns during treatment, highlighting a strong link between the immune landscape and the outcome of anti-PD1 therapy. Responders exhibited higher frequencies of circulating cDC1s, CD8^+^ T cells, and γδ2^+^ T cells in central memory (CM) stage. Notably, we observed a distinct remodeling of ICP expression profile, activation status and natural cytotoxicity receptor patterns of immune subsets during treatment. Anti-PD1 modulated DCs’ functionality and triggered deep changes in the functional orientation of T_conv_ and γδT cells.

**Discussion:**

Overall, our work provides new insights into the immunological landscape sustaining favorable clinical responses or resistance to first-line anti-PD1 therapy in melanoma patients. Such exploration participates in uncovering the mechanism of action of anti-PD1, discovering innovative predictive signatures of response, and paves the way to design pertinent combination strategies to improve patient clinical benefits in the future.

## Graphical Abstract

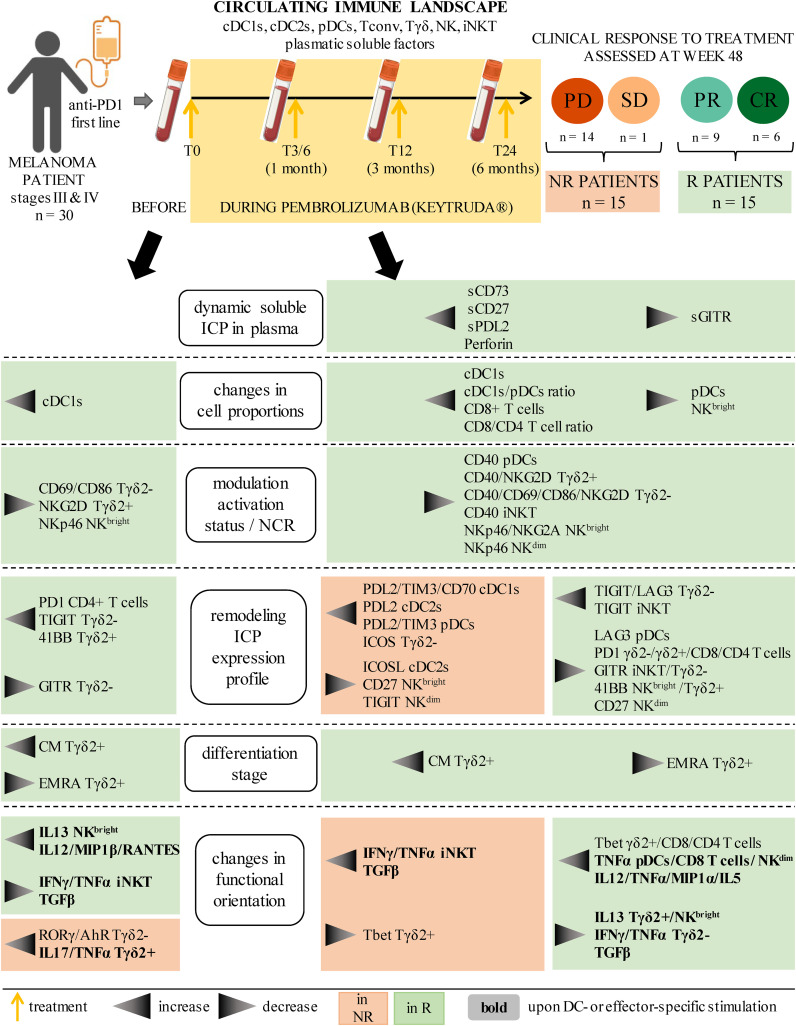

## 1 Introduction

Immune escape of melanoma is a challenge for clinicians, which could be reversed by immunotherapies. Among therapeutic options, immune checkpoint blockers (ICB) have revolutionized cancer treatments. In melanoma patients, response rates reached up to 45% for anti-PD1 monotherapy in therapeutic setting (1, 2), approaching 60% for anti-PD1/CTLA4 combotherapy (3). The clinical success of ICB deployed in melanoma strongly supports an efficient tumor control by the immune system on the long term. Despite unprecedented success and manageable safety profile, many patients endured immune mediated adverse effects (4), while others failed to respond to treatment (5). Currently, there are no specific biomarkers capable of predicting clinical response to ICB. The immune landscape and mechanisms leading to the failure of ICB in melanoma need to be further explored to improve outcomes and develop efficient and better tolerated immunotherapies (6).

Dendritic cells (DC) are active players in shaping anti-tumor immune responses (7) through different subsets harboring complementary functions. While cDC1 excel in cross-priming of tumor antigens to CD8^+^ T cells, cDC2 activate CD4^+^ T cells and drive Th1/Th17 responses. Plasmacytoid DC (pDC) participate in NK cell and CTL recruitment, and display direct cytotoxicity toward tumor cells (8). Melanoma hijacks DC subsets (9) by secreting immunosuppressive factors and immunomodulatory molecules (10), by exposing abnormal glycans (11, 12), or by causing metabolic stress interfering with DC differentiation, recruitment, maturation and function (10, 13). Furthermore, major modulations of OX40-L and ICOS-L on pDC led to the induction of Th2 and regulatory T cell (T_reg_) responses in melanoma patients (10). DC subsets harbor specific features, some of them displaying a high impact on clinical outcome (10, 14–16), and drive anti-tumor responses mainly through activation and priming of effectors. Alongside conventional (T_conv_) T cells, innate lymphoid cells (ILCs) (*e.g.*, NK cells) and innate-like T cells (ILTs) (*e.g.*, γδ T and iNKT cells) are crucial for cancer immune surveillance through their powerful cytolytic capacity against tumor cells and their cytokine secretion that potentiates other immune cells. ILCs and ILTs target a variety of non-peptidic antigens in an HLA-independent manner such as stress-induced ligands, glycolipids and/or phosphoantigens, and therapies exploiting their potential are consistently emerging (17, 18). NK, γδ T and iNKT cells infiltrate melanoma, yet phenotypic modulations and functional alterations were found both at tumor site and in blood (16, 19). Expression of ICP by these cells displays severe perturbations in patients (*e.g.*, dysregulation of TIM3, LAG3, 41BB, PD1, ICOS expression) (19). In addition, crosstalks between pDC and γδ T cells (16), and pDC and NK cells were altered, as well as the interrelations between DC subsets (14) in melanoma patients, highlighting the importance of immune cell networking. Moreover, crucial links exist between features of DC/ILC/ILT and patients’ clinical outcome. Indeed, the frequencies of intratumoral pDC (10, 20), circulating cDC1 (14), γδ T and γδ T expressing specific ICP (19), and the level of cytokine-secreting DCs (14) correlated with progression-free survival and overall survival of patients. Importantly, changes in circulation are informative on what occurs at the tumor site, as many modulations observed on tumor-infiltrating immune cells have been confirmed on circulating cells. Thus, DC subsets, T_conv_, NK, γδ T and iNKT cells, harbor crucial roles in anti-tumor responses and influence clinical outcomes. Yet, their involvement in response to ICB is poorly explored but of major interest due to their pivotal role in the control of tumor development.

Moreover, soluble immune checkpoints (sICP) –generated by alternative splicing or cleavage of the extracellular part by metalloproteinases– are of growing interest. Indeed, sICP can behave as decoy receptors or adjuvants and interfere with ICB efficacy (21). Associations between plasmatic sICP levels and response to ICB are emerging. Indeed, sPD-L1 and sLAG3 have been reported as prognosis factors of clinical evolution in several cancers (22); elevated sCTLA-4 levels correlated with clinical benefit in patients treated with Ipilimumab (23); sPD-L1 allowed to discriminate responder and non-responder non-small cell lung cancer patients receiving Nivolumab. Therefore, quantification of sICP in the plasma of ICB-treated patients could be promising to define predictive factors of response, but have not been studied yet in melanoma.

Several studies looked for predictive factors of response to ICB in cancer patients (6), especially in melanoma (23–27). Besides clinico-pathological elements and host factors such as the composition of the gut microbiota that revealed to be critical (28), current data mostly focused on tumor cells and T cells. Tumor mutational load or DNA repair were shown to be candidates, but difficult to apply in clinical routine. A high proportion of T cells or tumor cells expressing PD1 or PD-L1 were designated as predictive factors of response (29). The proportion of particular cell types (myeloid-derived suppressor cells [MDSC], lymphocytes, neutrophils, monocytes, NK), the diversity of TCRs, or the expression level of PD1 or TIM3 by T or NK cells have also been associated with response to ICB (30). Notably, the key role of DC subsets in response to ICB has been highlighted in melanoma mouse models. The presence of cDC1s within tumors (31) or CD5^+^ DCs (32) were essential for ICB efficacy. Thus, many parameters related to immune cell subsets could be interesting candidates to define predictive signatures of response to ICB, but remain to be investigated in human, and as part of a global immunological landscape.

Despite being standard of care and a field of intensive research, ICB still bring many scientific challenges and medical requirements. Identifying predictive signatures of response and deciphering mechanisms of response or non-response to treatment are critical points to optimize ICB efficacy and propose pertinent combination strategies. Altogether, available literature supports key involvement of DC subsets, T_conv_, NK, iNKT and γδ T cells in the control of melanoma tumor development, and that ICPs and cell networking may play a crucial role in the escape of melanoma from immune control. Such discoveries strongly support that these immune subsets could mediate ICB clinical efficacy or failure, and deserve to be further investigated. To explore these unmet challenges, we performed an immuno-monitoring on melanoma patients treated with first-line anti-PD1, before treatment (T0) and at weeks 3/6, 12, and 24 after administration. During treatment, we assessed in detail phenotypic and functional features of circulating cDC1, cDC2, pDC, NK (including CD56^bright^ and CD56^dim^ subsets), iNKT, γδ T (including δ2^+^ subset) and T_conv_ cells (including CD4^+^ and CD8^+^ T cells) by multi-parametric flow cytometry and Luminex. We depicted the frequency, activation status, NCR expression, characterized the expression of a large panel of ICP (together with quantification of sICP in plasma), assessed intra-nuclear transcription factors to depict Th profiles, and examined cell function by analyzing their ability to secrete cytokines/chemokines in response to stimulation. Such integration of the phenotypic and functional profiles together with clinical parameters provided a unique comprehensive understanding of the biological impact of anti-PD1 on crucial immune cell subsets participating in anti-tumor responses. Altogether, our work contributes in optimizing the therapeutic efficacy of ICB, better orienting therapeutic choices and designing pertinent combination strategies to improve patient clinical benefits in the future.

## 2 Materials and methods

### 2.1 Biobanking of melanoma patients’ blood samples

All procedures were approved by the Ethics committee of Grenoble University Hospital (CHUGA) and the French Blood Agency’s Institutional Review Board Committee (IRB), and accredited by the Ministry of Education and Research under the reference #AC-2020–3959 and approval #2023-A01722-43. Mandatory written informed consent was obtained from all patients prior to their participation in this study and samples were analyzed anonymously. All enrolled participants finished the study, i.e., there was no attrition. Sex is not a biological variable. Randomization was not used.

Blood samples were obtained from patients with unresectable (stage III) or metastatic (stage IV) melanoma (n=30) starting a first-line treatment with KEYTRUDA^®^ (anti-PD1; pembrolizumab) as monotherapy in curative setting in the Dermatology Department of CHUGA. Treatment was administrated by intravenous (IV) infusion at 200 mg every 3 weeks (wk) (n=21) or 400 mg every 6 wk (n=9), until progression or unacceptable toxicity. At each visit, a physical exam, vital signs, ECOG performance status, weight, concomitant medications, laboratory assessments and adverse events evaluation were performed. Tumor response assessments for efficacy were performed every 12 wk by a complete physical exam and tumor imaging by CT scan or MRI, and determined by clinicians using the Response Evaluation Criteria in Solid Tumors (RECIST 1.1 criteria). Patients were classified into two groups according to their clinical response after 48 weeks of treatment: responder (R): patients with a complete response (CR; disappearance of all target and non-target lesions) or a partial response (PR; at least a 30% decrease in the sum of all target lesions, taking reference at baseline and/or persistence of one or more non-target lesions); non-responder (NR): patients with a progressive disease (PD; at least a 20% increase in the sum of target lesions, taking as reference the smallest sum on study (including baseline) and an absolute increase of sum ≥ 5 mm or the appearance of one or more new lesions and/or the unequivocal progression of existing non-target lesions) or stable disease (SD; all patients that do no present a sufficient decrease to qualify for CR or PR nor a sufficient increase to qualify for PD).

Blood samples were collected from patients before treatment initiation (T0) and at each infusion (T3/6, T12 and T24 for weeks 3/6, 12 and 24 respectively) when possible. Plasmas were collected from blood samples upon centrifugation and stored at -80°C whereas, peripheral blood mononuclear cells (PBMCs) were isolated using Ficoll-Hypaque density gradient centrifugation (Eurobio) and stored frozen at -150°C. Clinical features of melanoma patients are summarized in **Supplementary Table 1**. For this cohort, the mean age was 72 years, median age 74 years, with min = 36 and max = 93 years. Experimenters were unaware (blinded) of group assignment and outcome assessment.

### 2.2 Flow cytometry immunophenotyping of patients’ PBMCs

Frozen PBMCs from four different time points for each patient (T0, T3/6, T12, T24) were thawed and stained in PBS 2% fetal calf serum (FCS) for 20 minutes at room temperature with multiple fluorochrome-labelled anti-human antibodies allowing the study of distinct immune cells (detailed gating strategy in **Supplementary Figure 1**). Dead cells were excluded with Live&Dead staining and Brilliant Stain buffer was used to limit staining artifacts caused by the utilization of several antibodies conjugated with fluorescent polymer dyes. After washing and fixation using BD FACS™ lysing solution, stained cells were analyzed using LSRII Flow Cytometer and FACSDiva software 9. Isotype controls were used to differentiate positive cells from nonspecific background staining (CD45^+^ cells were also used to determine the positivity threshold). To ensure quality control, standardization of the fluorescence intensities was performed using cytometer setup and tracking beads (CST).

#### 2.2.1 Phenotyping of DC subsets

The combination of anti-human CD45, Lineage cocktail, HLA-DR and CD11c antibodies depicted DC subsets, while antibodies directed against BDCA1/CD1c, BDCA2/CD303 and BDCA3/CD141 allowed to define cDC2s, pDCs and cDC1s respectively. We used the same fluorophore for BDCA2 and BDCA3 antibodies since the corresponding DC subsets were discernable by different intensities of labeling. To study the basal activation status and ICP expression of DC subsets, CD80, CD86, CD40, PD-L1, PD-L2, LAG-3, 41BB-L, ICOS-L, GITR-L, OX40-L, TIM-3 and CD70 fluorophore-labeled anti-human antibodies were used.

#### 2.2.2 Phenotyping of effector immune cells

Effector immune cells were depicted by using the combination of anti-human CD45, CD3, CD56, CD8, TCRγδ, Vδ2 TCR and TCR Vα24-Jα18 (iNKT cell) fluorophore-labeled antibodies. The basal activation status, ICP and/or KIR/NCR expression of conventional T cells (CD4^+^ or CD8^+^ T cells), γδ T cells (δ2^+^ or δ2^-^ γδ T cells), iNKT and NK cells (CD56^bright^ or CD56^dim^) were assessed using fluorophore-labeled anti-human CD40, CD86, CD69, CD25, GITR, PD1, TIM-3, 41BB, CTLA-4, LAG-3, ICOS, OX40, CD27, TIGIT, NKp30, NKp44, NKp46, NKG2A, NKG2C and NKG2D antibodies. Differentiation status of T cells (effector memory cells re-expressing CD45RA [EMRA], effector memory [EM], central memory [CM], and naïve [N] cells) was also analyzed using anti-human CD27 and CD45RA fluorophore-labeled antibodies.

#### 2.2.3 Intranuclear transcription factor staining within effector immune cells

For intranuclear transcription factor characterization, PBMCs were first stained for surface markers allowing to depict effector immune cells (anti-human CD45, CD3, CD8, TCRγδ, Vδ2 TCR and TCR Vα24-Jα18 fluorophore-labeled antibodies) (15min at room temperature) and then fixed and permeabilized using the eBioscience™ FoxP3/Transcription Factor Staining Buffer Set and according to manufacturer’s instructions (Invitrogen). Intranuclear transcription factor staining was then performed using anti-human RORγt, GATA-3, AHR, FoxP3 and T-bet fluorophore-labeled antibodies (20min at room temperature). Cells were then washed and fixed in BD FACS™ lysing solution.

### 2.3 Functional analysis of patients’ PBMCs in response to *in-vitro* stimulation

Cultures were performed in RPMI 1640 medium supplemented with 1% nonessential amino acids, 100 μg mL^-1^ gentamicin, 10% FCS and 1 mmol L^-1^ sodium pyruvate at 37°C, 5% CO2. For cytokine production assessment, PBMCs were cultured whether at 2x10^6^ cells mL^-1^ for DC subsets or at 0.5x10^6^ cells mL^-1^ for effector immune cells. DC subsets were stimulated with either Polyinosine-polycytidylic acid (PolyI:C; 30 µg mL^-1^), Resiquimod (R848; 1µg mL^-1^), Class A CpG oligonucleotide (CpGA ODN 2336; 1,5 µM) or the mixture of the three TLR ligands (TLR-L, a combination of polyI:C, R848 and CpGA). Effector cells were cultured in the presence of IL-2 (0.1 IU mL^-1^) and stimulated with either Phorbol 12-myristate 13-acetate (PMA; 20 ng mL^-1^) and Ionomycin calcium salt (Iono; 1 µg mL^-1^), or anti-CD3/CD28 antibodies, (E)-1-Hydroxy-2-methyl-2-butenyl-4-pyrophosphate lithium salt (HMB-PP; 200 nM), IL-12/IL-18 (50 ng mL^-1^), α-Galactosylceramide (αGalCer; 100 ng mL^-1^) or a mixture of several activators (CD3/CD8, HMB-PP, IL-12/IL-18 and αGalCer). Immune cells’ functionality was then assessed by flow cytometry (intracellular cytokine staining) and/or cytokine secretion measurements by LUMINEX technology.

#### 2.3.1 Intracellular cytokine staining by flow cytometry

Following PBMC incubation with immune cell activators, 1 µg mL^-1^ of Brefeldin A was added after 1h or 2h30 for DC subsets or effector immune cells, respectively. Later on, cells were stained for surface markers allowing to depict DC subsets (anti-human CD45, Lineage cocktail, HLA-DR, CD11c, BDCA1, BDCA2 and BDCA3) or effector cells (anti-human CD45, CD3, CD8, CD56, TCRγδ, Vδ2 TCR and TCR Vα24-Jα18) for 15min at room temperature and then fixed and permeabilized using the Cytofix/Cytoperm™ Plus Fixation/Permeabilization Solution Kit and following manufacturer’s instructions (BD Biosciences). Dead cells were excluded with Live&Dead staining and Brilliant Stain buffer was used to limit staining artifacts caused by the utilization of several antibodies conjugated with fluorescent polymer dyes. Intracellular cytokine staining of DC subsets was performed using anti-human fluorophore-labeled antibodies IFN-α, TNF-α and IL-12p40/p70 and unconjugated IFN-λ1 antibody stained using Mix-n-Stain™ CF^®^488A dye for 20min at room temperature. On the other hand, intracellular cytokine staining of effector immune cells was done using anti-human fluorophore-labeled IL-13, IL-17, IFN-γ, TNF-α and IL-10 antibodies for 20min at room temperature. After washing and fixation, stained cells were analyzed using LSRII Flow Cytometer and FACSDiva software 9. Isotype controls were used to differentiate positive cells from nonspecific background staining (CD45^+^ cells were also used to determine the positivity threshold). To ensure quality control, standardization of the fluorescence intensities was performed using cytometer setup and tracking beads (CST).

#### 2.3.2 Cytokine secretion by LUMINEX technology

To study cytokine secretion by patients’ PBMCs after *in vitro* stimulation, samples were cultured at 1x10^6^ cells mL^-1^ for 20 to 21 hours with or without a single or a mixture of TLR-L or immune activators previously described. Culture supernatants were then collected and stored at -20°C until use. For DCs’ secretome assessment, fractalkine (CX3CL1), I-TAC (CXCL11), IFN-α, IFN-β, IL-1α, IL-1β, IL-6, IL-8 (CXCL8), IL-10, IL-12p70, IL-23, IL-29 (IFN-λ1), IP-10 (CXCL10), MCP-1 (CCL2), MIG (CXCL9), RANTES (CCL5), TNF-α, MDC, MIP-1α (CCL3), MIP-1β (CCL4), TARC (CCL17) and TGF-β1 were measured in supernatants from PBMC cultures with TLR-L (a combination of polyI:C, R848 and CpGA). To evaluate effector cells’ secretomes, the following soluble factors were measured in supernatants from PBMC cultures with immune activators: IFN-γ, IL-4, IL-5, IL-10, IL-13, IL-17A, IL-22, TNF-α and TGF-β1. The dosages were conducted following manufacturer’s instructions and measured by LUMINEX technology using MAGPIX^®^200 Instrument with xPONENT^®^ software.

### 2.4 Cytokine and chemokine assessment in patient’s plasma

To evaluate patient’s cytokine and chemokine basal status, collected plasmas were thawed and the following soluble factors were measured for each patient: Arginase-1, B7-H6, BTLA, CD27, CD28, CD47 (IAP), CD48 (BLAST-1), CD73 (NT5E), CD80, CD96 (Tactile), CD134 (OX40), CD137 (4-1BB), CD152 (CTLA4), CD276 (B7-H3), E-Cadherin, GITR, HVEM, ICOS Ligand (B7-H2), IDO, LAG-3, MICA, MICB, Nectin-2, PD1, PD-L1, PD-L2, PVR, Perforin, S100A8/A9, Siglec-7, Siglec-9, TIM-3, TIMD-4, ULBP-1, ULBP-3, ULBP-4 and VISTA (B7-H5). The dosages were conducted following manufacturer’s instructions and measured by LUMINEX technology using MAGPIX^®^200 Instrument with xPONENT^®^ software.

### 2.5 Statistical analysis

Statistical analyses were done using GraphPad Prism software and applying non-parametric Kruskal-Wallis test for inter-group comparisons and/or Wilcoxon matched-pairs signed rank test with Bonferroni correction for intra-group analyses, or Spearman test for correlations. Data are shown as medians, and significance threshold was set at *P* < 0.0167. Euclidean distance-based hierarchical clustering (heat maps), PCA, correlation matrix and radar plots were performed using the RColorBrewer (to select color palettes), gplots (function heatmap.2), missMDA (function imputePCA), FactoMineR (function PCA), ggbiplot (function ggbiplot), factoextra (functions fviz_pca_var and fviz_contrib), corrplot (function corrplot), psych (function corr.test) and fmsb (function radarchart) packages of the R software.

## 3 Results

### 3.1 Study design

Blood samples were collected from melanoma patients with unresectable (stage III) or metastatic (stage IV) melanoma (n=30) starting a first-line treatment with KEYTRUDA^®^ (anti-PD1; pembrolizumab) as monotherapy in a curative setting, at treatment initiation (T0), and when possible at several infusions (T3/6, T12 and T24 for weeks 3/6, 12 and 24, respectively). PBMC and plasma were retrieved and stored until use. Patients were classified into different groups according to their clinical response to immunotherapy after 48 weeks of treatment. Their clinical features are reported in **Supplementary Table 1**. Patients with complete response (CR; n=6) had a disappearance of all target and non-target lesions, while patients with a partial response (PR; n=9) showed at least a 30% decrease in the sum of all target lesions when taking the baseline as reference and/or persistence of non-target lesions. Patients with a progressive disease (PD; n=14) had at least a 20% increase in the sum of target lesions when taking as reference the smallest sum on study (including baseline), and an absolute increase of sum ≥ 5mm or the appearance of new lesions. Patients who did not present a sufficient decrease or an increase of target lesions were grouped and classified as stable disease (SD; n=1).

We explored in each patient sample ten distinct immune cell types for which features were previously shown to be pivotal in dictating the clinical outcome of melanoma patients using a specific multi-parametric flow cytometry approach (**Supplementary Figure 1A**). On one hand, DC subsets were defined amongst alive CD45^+^Lin^–^HLA-DR^+^ cells as CD11c^dim^BDCA3^+^ cDC1s, CD11c^+^BDCA1^+^ cDC2s and CD11c^–^BDCA2^+^ pDCs. On the other hand, effector cells were depicted amongst alive CD45^+^ cells as follows: CD3^–^CD56^+^ for NK cells (NK^bright^ and NK^dim^ depending on CD56 expression levels); CD3^+^Vδ2TCR^+^ for γδ2^+^T cells; CD3^+^TCRγδ^+^Vδ2TCR^–^ for γδ2^–^T cells (γδ2^+^T cells appears TCRγδ negative due to competition between the two antibodies targeting the δ chain, **Supplementary Figure 1B**); CD3^+^TCRγδ^–^TCRVα24-Jα18^+^ for iNKT cells; CD3^+^TCRγδ^–^TCRVα24-Jα18^–^CD8^+^ for CD8^+^ T cells; and CD3^+^TCRγδ^–^TCRVα24-Jα18^–^CD8^–^ for CD4^+^ T cells. We examined cell proportions, basal activation status, ICP and/or NCR expression and functionality for every immune cell subset studied. Memory T-cell differentiation stage and Th profile were also studied for γδ2^+^T, γδ2^–^T, CD8^+^ T and CD4^+^ T cells. In addition, soluble factors were explored in cell supernatants after external stimulation (cytokines, chemokines) and in plasma collected from patients (sICP, immune regulators, adhesion molecules, lectin receptors) by ProcartaPlex^®^ dosages using Luminex.

### 3.2 Higher proportions of circulating cDC1s and CD8^+^ T cells were found before and/or during treatment in melanoma patients responding to immunotherapy

**Figure 1:**
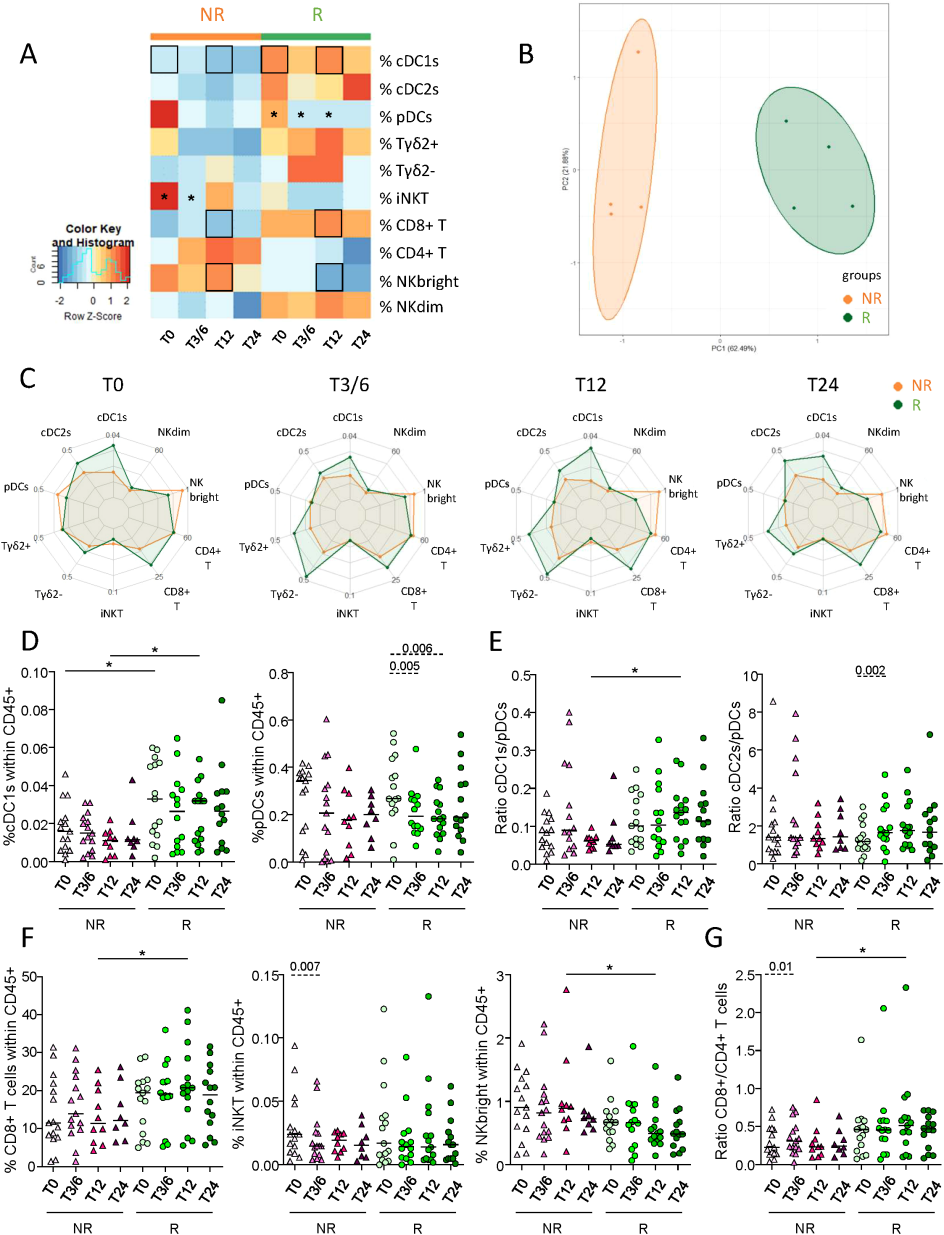
Distinct frequencies of circulating cDC1s, CD8^+^ T and NK^bright^ cells in melanoma patients responding to anti-PD1 therapy compared to non-responding patients. By using a multi-parametric flow cytometry approach, the frequencies of ten immune cell populations were evaluated in the circulation of melanoma patients treated with anti-PD1 and patients were later compared depending on their clinical response to the treatment. **(A)** Heat map based on the median frequencies of each of the ten immune subpopulations studied in patient’s blood (NR, non-responders; R, responders) at different time points of the treatment (T0, T3/6, T12, T24). Statistically significant comparisons between patient groups (inter-groups) are shown as black squares (non-parametric Kruskal-Wallis test), and the ones between T0 and another time point in a specific patient group (NR or R patient group, intra-group) are illustrated as black stars (Wilcoxon matched-pairs signed rank test with Bonferroni correction). **(B)** PCA based on the median frequencies of the immune populations studied in patient’s blood (NR or R patients) at different time points of the treatment. **(C)** Radar plots showing the median frequencies of the ten immune cell populations studied (within CD45^+^ cells) in non-responders (NR, orange line) and responders (R, green line) at different time points upon treatment initiation. **(D, E)** Frequencies of cDC1s and pDCs within CD45^+^ cells **(D)** and ratios of cDC1s/pDCs and cDC2s/pDCs **(E)** in NR (triangles) and R (circles) melanoma patients at different time points of the treatment (n = 8 to 15 per group). **(F, G)** Frequencies of CD8^+^ T, iNKT and NK^bright^ cells within CD45^+^ cells **(F)** and ratio of CD8^+^/CD4^+^ cells **(G)** in NR (triangles) and R (circles) melanoma patients at different time points of the treatment (n = 8 to 15 per group). **(D–G)** Bars indicate median. P-values were calculated using non-parametric Kruskal-Wallis test (straight lines) or Wilcoxon matched-pairs signed rank test with Bonferroni correction (dashed lines). Only significant statistics are displayed on graphs. *P ≤ 0.05.

To investigate the relevance of circulating DC subsets (cDC1s, cDC2s and pDCs) and effector cells (γδ2^+^T, γδ2^–^T, iNKT, CD4^+^ T, CD8^+^ T, NK^bright^ and NK^dim^ cells) in the response to anti-PD1, we first assessed the frequencies of these immune cells in the blood of melanoma patients before and during the treatment (T0, T3/6, T12, T24). Given the limited number of patients in some groups, we regrouped patients as non-responders (NR; PD + SD; n=15) or responders (R; PR + CR; n=15) throughout the study. Heat map visualization and PCA analysis based on median proportions of circulating immune cells of NR or R patients brought forward different cell frequency patterns and allowed to distinguish the two response groups (**Figures 1A, B**), mostly by cDC1 and CD8^+^ T cell frequencies (**Supplementary Figure 2A**). Radar plot illustrations also highlighted variations of circulating immune cell proportions both between the response groups (inter-group) at different cures (also referred as time points) and within each group (intra-group) during the duration of the treatment (**Figure 1C**; **Supplementary Figure 2B**). For circulating DC subsets, the frequency of cDC1s was higher in R when compared to NR patients at T0 and T12, and these levels were maintained throughout the duration of the treatment for responders. Interestingly, in both groups, we noticed a drop in pDC frequency after the first cure, which reached significance only in responders (**Figure 1D**). The cDC1/pDC ratio was also higher in R when compared to NR patients at T12, and we noticed an increase of the cDC2/pDC ratio between T0 and T3/6 in responders (**Figure 1E**), probably due to the decrease of pDCs since cDC2s’ proportions remained unchanged (**Supplementary Figure 2F**). Regarding immune effector cells, the frequency of CD8^+^ T cells as well as the CD8^+^/CD4^+^ T cells ratio increased, while the proportion of NK^bright^ cells decreased in R when compared to NR patients at T12 (**Figures 1F, G**). The proportion of iNKT cells also decreased after the beginning of the treatment in non-responders (comparison between T0 and T3/6; **Figure 1F**). Besides, proportions of circulating CD4^+^ T, γδ2^+^T, γδ2^-^T and NK^dim^ cells remained unchanged both between groups and within each group during the course of the treatment (**Supplementary Figure 2C**). To assess interrelations between immune cell populations, we performed statistical Spearman’s correlations between the frequencies of the immune subpopulations studied, and found no significant differences between NR and R patients (**Supplementary Figure 2D**). Thus, these results indicate that responder melanoma patients had higher frequencies of circulating cDC1s and CD8^+^ T cells before and/or during anti-PD1 treatment when compared to non-responders.

### 3.3 Melanoma patients not responding to immunotherapy exhibited higher levels of circulating PD-L2-, CD70- and/or TIM3-expressing DC subsets during anti-PD1 treatment in comparison with responders

**Figure 2:**
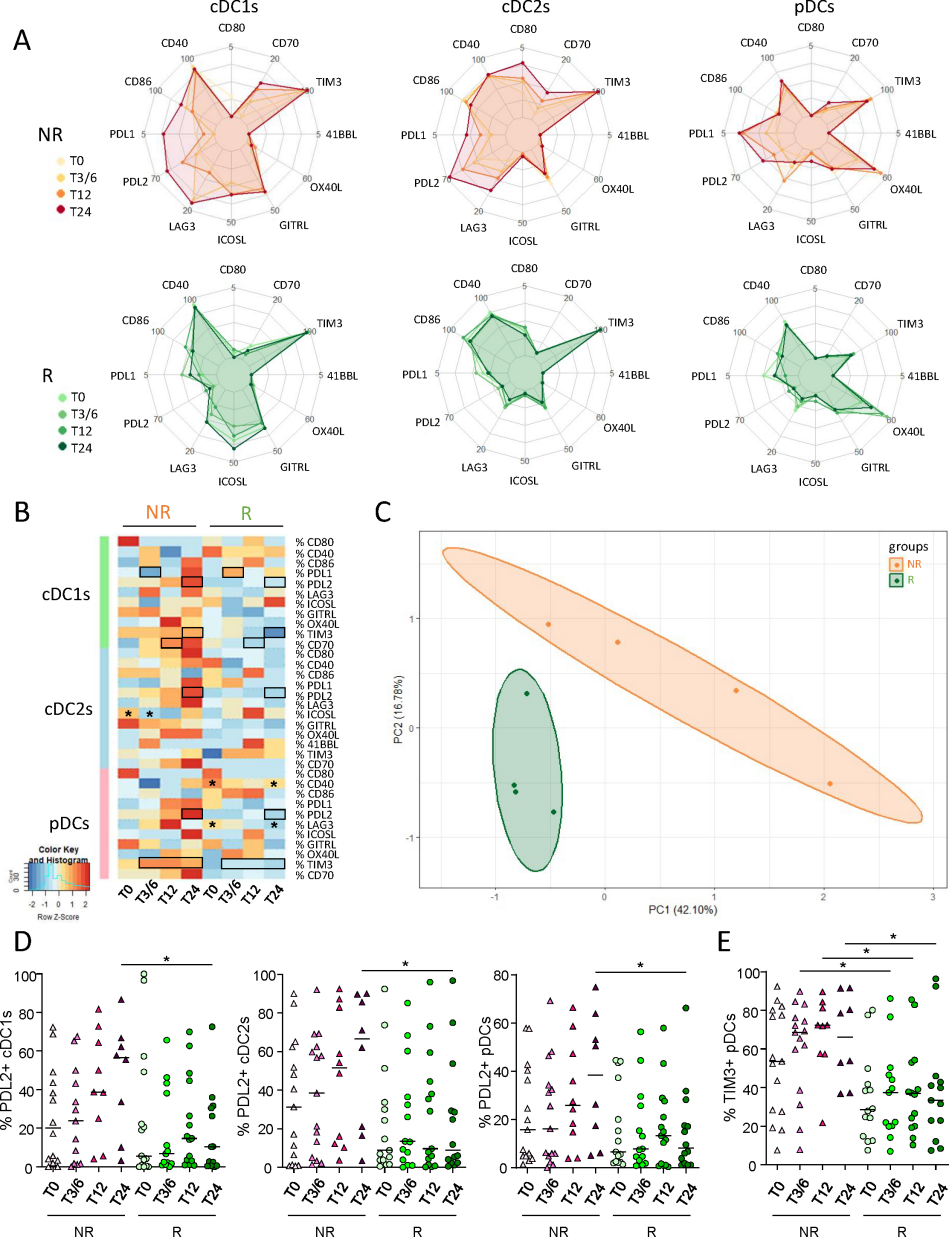
Lower levels of PD-L2- and/or TIM3-expressing DC subsets in the circulation of responder compared to non-responder melanoma patients during anti-PD1 treatment. To examine the relevance of DC subsets’ status in the response to anti-PD1, we assessed the basal activation state and ICP expression profile on circulating DC subsets from melanoma patients treated with immunotherapy using multi-parametric flow cytometry. **(A)** Radar plots showing the median proportions of cDC1s-, cDC2s- and pDCs-expressing activation markers and ICP (within the corresponding cell subset) at different time points of the treatment (T0, T3/6, T12, T24) in non-responder (NR; upper panels) and responder (R; lower panels) patients. **(B)** Heat map based on the median frequencies of cDC1s-, cDC2s- and pDCs-expressing activation markers and ICP in patient’s blood (NR or R patients) at different time points of the treatment. Statistically significant comparisons between patient groups (inter-groups) are showed as black squares (non-parametric Kruskal-Wallis test), and the ones between T0 and another time point within a specific patient group (NR or R patient group, intra-groups) are illustrated as black stars (Wilcoxon matched-pairs signed rank test with Bonferroni correction). **(C)** PCA based on the median frequencies of cDC1s-, cDC2s- and pDCs-expressing activation markers and ICP in patient’s blood (NR or R patients) at different time points of the treatment. **(D, E)** Frequencies of PD-L2^+^ cDC1s, cDC2s or pDCs **(E)** and TIM3^+^ pDCs **(E)** in NR (triangles) and R (circles) melanoma patients at different time points of the treatment (n = 8 to 15 per group). Bars indicate median. Frequencies are indicated within the corresponding cell subset. P-values were calculated using non-parametric Kruskal-Wallis test (straight lines) or Wilcoxon matched-pairs signed rank test with Bonferroni correction (dashed lines). Only significant statistics are shown on graphs. *P ≤ 0.05.

To examine the importance of DC phenotype in the response to anti-PD1, we assessed the basal activation state and ICP expression profile of circulating DC subsets from immunotherapy-treated melanoma patients using flow cytometry (**Supplementary Figure 3A**). Radar plot underlined distinct phenotypic profiles of circulating DC subsets both inter-group (**Supplementary Figure 3B**) and intra-group during the duration of the treatment (**Figure 2A**). In addition, heat map and PCA analysis based on the median proportions of DC subsets expressing activation markers and ICP of patients (NR, R) also highlighted different patterns according to response to treatment. This distinguished the two response groups (**Figures 2B, C**) mostly based on PD-L2 and CD70 expression by DC subsets (**Supplementary Figure 3C**). Overall, we observed tendencies toward increased proportions of PD-L2-expressing DC subsets (**Figure 2D**), together with cDC1s and pDCs expressing CD70 and/or TIM3 in non-responders during the course of anti-PD1 therapy, contrary to responders whose frequencies remained stable (**Figure 2E**; **Supplementary Figure 3D**). Such evolution contributed to significantly higher circulating frequencies of PD-L2-expressing cDC1s, cDC2s and pDCs, TIM3-expressing cDC1s and pDCs, and of CD70-expressing cDC1s in NR compared to R patients at T3/6, T12 and/or T24 (**Figure 2E**; **Supplementary Figure 3D**). In addition, intra-group comparisons revealed decreased frequencies of ICOS-L^+^ cDC2s after the beginning of the treatment in non-responders at T3/6, as well as of proportions of LAG3- or CD40-expressing pDCs during treatment in responders at T24 compared to baseline (**Supplementary Figure 3E**). Furthermore, we performed Spearman’s correlations to assess the association between PD-L1/-L2 expression and other ICPs expressed by DC subsets in patients (NR, R) before the start of anti-PD1 treatment. The expression of PD-L1 and/or PD-L2 on DC subsets positively correlated with the expression of LAG3, CD70, OX40L and/or TIM3 in NR patients, while only positive correlations between PD-L1^+^ cDC2s and LAG3^+^ or OX40L^+^ cDC2s were preserved in responders before anti-PD1 (**Supplementary Figures 4A–C**). Thus, these results show that non-responders had higher proportions of circulating PD-L2^+^, CD70^+^ and/or TIM3^+^ DC subsets during anti-PD1 treatment than responders.

### 3.4 Responder and non-responder melanoma patients displayed differences in the activation status, ICP and NCR expression profiles of circulating immune effector cells before and during anti-PD1 treatment

**Figure 3:**
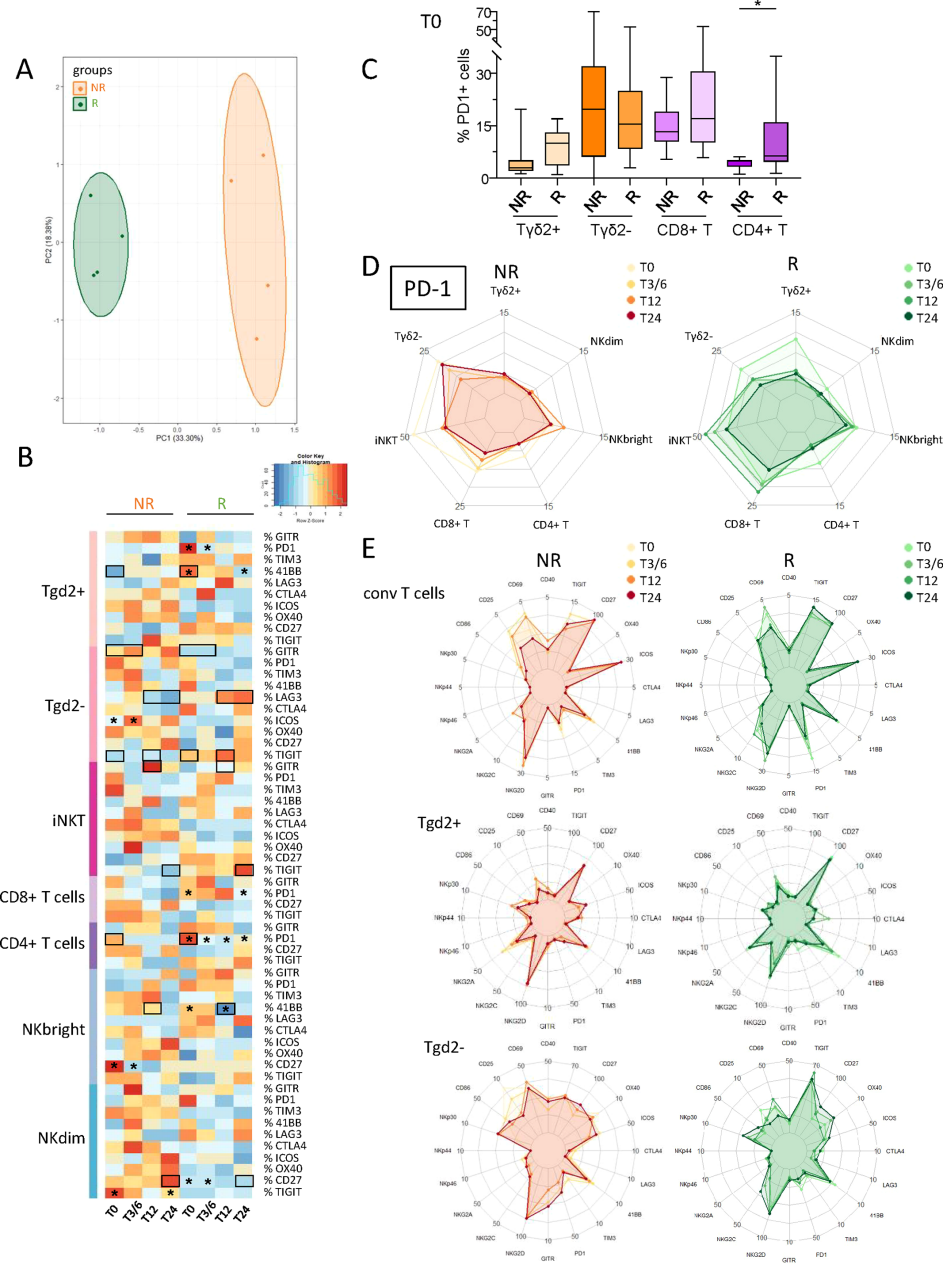
Circulating immune effector cells from melanoma patients displayed distinct ICP profiles according to their clinical response to anti-PD1 treatment, and during the therapy process. In order to decipher the importance of effector cells’ status for the response to immunotherapy, we investigated the activation status and ICP expression profile on circulating immune effector cells from melanoma patients following anti-PD1 treatment using multi-parametric flow cytometry, and compared the cell features according to patient clinical response to the treatment. **(A)** PCA based on the median frequencies of effector cells-expressing activation markers and ICP (within the corresponding cell subset) in patient’s blood (NR, non-responders; R, responders) at different time points of the treatment (T0, T3/6, T12, T24). **(B)** Heat map based on the median frequencies of effector cells-expressing ICP in patient’s blood (NR and R patients) at different time points of the treatment. Statistically significant comparisons between patient groups (inter-groups) are showed as black squares (non-parametric Kruskal-Wallis test), and the ones between T0 and another time point in a specific patient group (NR or R patient group, intra-group) are illustrated as black stars (Wilcoxon matched-pairs signed rank test with Bonferroni correction). **(C)** Box and whiskers plot illustrating the proportions of circulating PD1–expressing cells (γδ2^+^T, γδ2^–^T, CD8^+^ T and CD4^+^ T cells) in NR and R melanoma patients before immunotherapy (T0; n = 14 to 15). Bars indicate median. P-values were calculated using non-parametric Kruskal-Wallis test (straight lines) or Wilcoxon matched-pairs signed rank test with Bonferroni correction (dashed lines). Only statistically significant comparisons are displayed on graphs. *P ≤ 0.05. **(D)** Radar plots showing the median proportions of PD1^+^ effector cells in NR and R melanoma patients at different time points of the treatment. **(E)** Radar plots showing the median proportions of effector cells expressing the studied activation markers and ICPs (T_conv_, γδ2^+^T and γδ2^-^T cells) in NR (left panels) and R (right panels) melanoma patients at different time points of the treatment. Frequencies are indicated within the corresponding cell subset.

To investigate the potential differences between NR and R patients to anti-PD1, the activation status, ICP and NCR expression profiles of circulating effector cells from melanoma patients were investigated using flow cytometry (**Supplementary Figure 5A**). PCA analysis based on phenotypic features of effector cells allowed the clustering of patient groups (NR versus R) at different times of the treatment (T0, T3/6, T12, T24), mostly driven by the status of γδ2^+^T, γδ2^-^T and NK^dim^ cells (**Figure 3A**; **Supplementary Figure 5B**). Heat maps also illustrated distinct patterns of activation status and NCR expression (**Supplementary Figure 5C**) as well as ICP expression profile (**Figure 3B**) on effector cells in R compared to NR patients before and during anti-PD1 treatment. We first focused on PD1, as this molecule is targeted by the therapy and expression of its ligands (PD-L1, and mostly PD-L2) was perturbed on DC subsets. Before anti-PD1 treatment initiation, higher frequencies of PD1-expressing CD4^+^ T cells (and potentially γδ2^+^T) were found in the blood of R compared to NR patients (**Figure 3C**). During treatment, several effector cell subsets (γδ2^+^T, γδ2^–^T and T_conv_ cells, the latter divided into CD8^+^ or CD4^+^ T cells) from R patients endured a significant decrease in PD1 expression (**Figure 3D**; **Supplementary Figure 5D**). When assessing other ICPs, activation and NCR markers on circulating effector cells, our study revealed both inter-group differences according to response to treatment and modulations of patterns during the course of the treatment (**Figure 3E**; **Supplementary Figures 5E, F**). Before anti-PD1 treatment, proportions of circulating 41BB-expressing γδ2^+^T and TIGIT-expressing γδ2^–^T cells were higher in R compared to NR patients, whereas proportions of GITR^+^ γδ2^–^T cells were decreased (**Supplementary Figure 5G**). During treatment, higher proportions of 41BB^+^ T_conv_ cells, LAG3^+^ γδ2^–^T and TIGIT^+^ iNKT cells were observed in R compared to NR patients, while frequencies of GITR-expressing γδ2^–^T and iNKT, 41BB-expressing NK^bright^ and CD27-expressing NK^dim^ cells were decreased in responder melanoma patients (**Supplementary Figure 5G**). Regarding intra-group comparisons, we observed in NR patients decreased proportions of TIGIT^+^ NK^dim^ and CD27^+^ NK^bright^, while proportions of 41BB^+^ γδ2^+^T and NK^bright^ cells and CD27^+^ NK^dim^ cells significantly decreased in responders (**Supplementary Figure 5G**). Correlations of PD1 expression with other ICPs on effector cells before treatment initiation (T0) showed a positive correlation between PD1 and GITR expression on γδ2^–^T cells derived from non-responders, while a negative correlation was observed between PD1 and TIGIT expression on circulating CD8^+^ T cells in responders (**Supplementary Figure 5H**). Concerning activation status, the levels of CD40-, CD86- and/or CD69-expressing γδ2^+^T, γδ2^–^T and iNKT cells were lower in the blood of R when compared to NR patients before and/or during anti-PD1 treatment (**Supplementary Figure 5I**). Regarding NCR expression, we observed a decreased level of NKG2D on γδ2^+^T and lower proportions of circulating NKp46-expressing NK^bright^ cells in R compared to NR patients before treatment (**Supplementary Figure 5J**). Furthermore, there were lower levels of NKG2D or NKG2A on γδ2^+^T and NK^bright^ cells respectively during treatment, and of NKp46-expressing NK^dim^ cells, whereas NKG2C-expressing iNKT increased in R compared to NR patients (**Supplementary Figures 5J**). Regarding intra-group comparisons, we observed during anti-PD1 treatment a decrease of NKp46-expressing NK^bright^ cells in NR patients, while there was an increase of NKG2C^+^ γδ2^–^T cells specifically in R patients (**Supplementary Figure 5J**). Thus, these data highlight distinct activation, ICP and NCR patterns on circulating effector cells in responder and non-responder melanoma patients during anti-PD1 treatment.

### 3.5 Soluble factors found in plasma of melanoma patients did not allow the distinction of responders from non-responders before the beginning of anti-PD1 treatment

Soluble isoforms of ICP are generated by alternative splicing or cleavage of extracellular parts by metalloproteinases. Such molecules can behave as decoy receptors or adjuvants and potentially interfere with ICB efficacy. In this context, we analyzed 37 soluble factors (especially sICP) in the plasma of melanoma patients before and during the course of anti-PD1 treatment by using Luminex. Heat map visualization based on median levels of soluble factors found on the plasma of NR and R patients did not show major inter-group differences according to the patient response to the treatment (**Supplementary Figure 6A**), except for an increase in soluble CD73 in R compared to NR patients at T24 (**Supplementary Figure 6B**). Besides, intra-group analyses highlighted increases of soluble CD27, PD-L2 and perforin, and a decrease of soluble GITR during anti-PD1 treatment in R patients exclusively (**Supplementary Figure 6B**).

### 3.6 Higher frequencies of circulating γδ2^+^T cells in the central memory stage (and lower frequencies in the EMRA stage) were observed in responders compared to non-responder melanoma patients before and during anti-PD1 treatment

**Figure 4:**
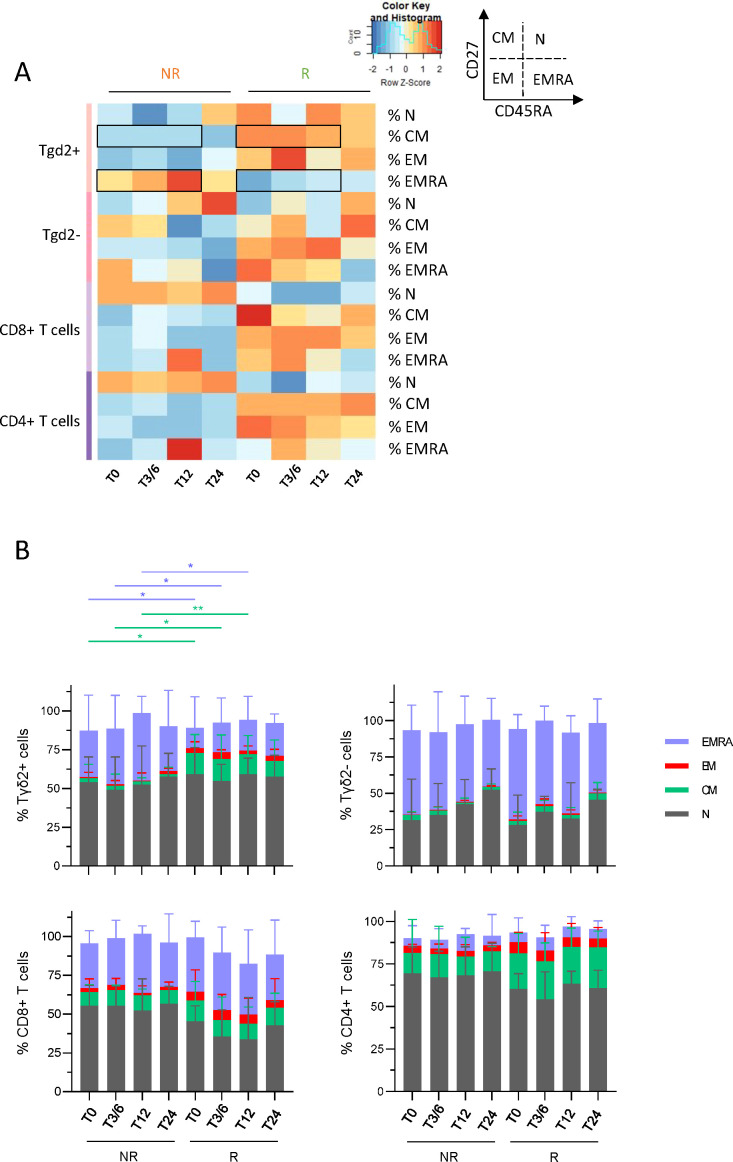
Melanoma patients responding to immunotherapy owned higher frequencies of circulating γδ2^+^T cells in central memory stage and lower frequencies in EMRA stage before and during the treatment when compared to non-responders. To examine whether T-cell differentiation stage differed depending on the patient’s clinical response to immunotherapy, the frequencies of N, CM, EM and EMRA populations of γδ2^+^T, γδ2^-^T, CD8^+^ T and CD4^+^ T cells were assessed in melanoma patients undergoing anti-PD1 treatment by multi-parametric flow cytometry. **(A)** Heat map based on the median proportions of T-cell populations according to their differentiation stage (N, naive; CM, central memory; EM, effector memory; EMRA, effector memory re-expressing CD45RA) of γδ2^+^T, γδ2^-^T, CD8^+^ T and CD4^+^ T cells in patient’s blood (NR, non-responders; R, responders) at different time points of the treatment (T0, T3/6, T12, T24). Statistically significant comparisons between patient groups (inter-groups) are showed as black squares (non-parametric Kruskal-Wallis test). **(B)** Bar plots illustrating the median frequencies of N, EM, CM and EMRA populations within γδ2^+^T, γδ2^-^T, CD8^+^ T and CD4^+^ T cells in NR and R melanoma patients at different time points of the treatment (n = 8 to 15 per group). Frequencies are indicated within the corresponding cell subset. P-values were calculated using non-parametric Kruskal-Wallis test (straight lines) or Wilcoxon matched-pairs signed rank test with Bonferroni correction (dashed lines). Only significant statistics are displayed on graphs. *P ≤ 0.05, **P ≤ 0.01.

To examine whether T-cell differentiation stage fluctuated depending on the patient’s clinical response to immunotherapy, the frequencies of differentiation stages (N: naive; CM: central memory; EM: effector memory; EMRA: effector memory re-expressing CD45RA) of γδ2^+^T, γδ2^–^T, CD8^+^ T and CD4^+^ T cells were evaluated in melanoma patients undergoing anti-PD1 treatment using flow cytometry. Heat map visualization and PCA analysis based on the median frequencies of T-cell differentiation stages derived from the blood of NR and R patients at different time points (T0, T3/6, T12, T24) illustrated distinct patterns of differentiation stage allowing the clustering of both response groups, mostly driven by differences on γδ2^+^T cells (**Figure 4A**; **Supplementary Figure 7**). Before and during anti-PD1 treatment, frequencies of circulating γδ2^+^T cells in the CM stage were higher in R compared to NR patients, while there were fewer in the EMRA stage (**Figure 4B**). Such picture was also depicted for CD8^+^ and CD4^+^ T cells, even though it was not significant.

### 3.7 Higher levels of IL-12p70, TNF-α and MIP-1β and lower levels of TGF-β1 were found in the supernatants derived from PBMC of melanoma patients responding to immunotherapy upon DC-specific stimulation

**Figure 5:**
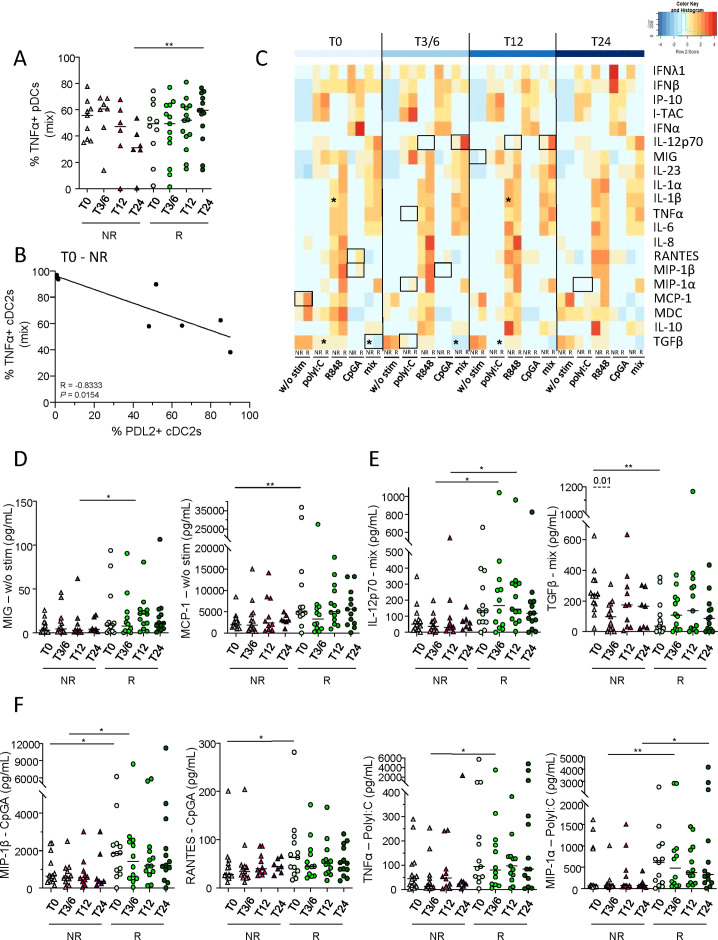
Higher levels of IL-12p70, TNF-α and MIP-1β and lower levels of TGF-β1 were found in supernatants derived from PBMC of melanoma patients responding to immunotherapy when compared to non-responders upon DC-specific stimulation. To investigate the functionality of DC subsets in melanoma patients following anti-PD1 treatment, cytokine/chemokine production and secretion by DCs after TLR stimulation were assessed respectively by intracellular cytokine staining using flow cytometry and ProcartaPlex dosages of culture supernatants using Luminex. **(A)** Frequency of circulating TNF-α^+^ pDCs, after 5h culture with TLR-L mixture (mix; combination of polyI:C, R848 and CpGA), in non-responder (NR; triangles) and responder (R; circles) patients at different time points of the treatment (T0, T3/6, T12, T12; n = 6 to 14 per group). **(B)** Spearman’s correlation between the proportions of circulating TNF-α^+^ cDC2s after mix stimulation and of PD-L2^+^ cDC2s in non-responder melanoma patients before immunotherapy (T0-NR; n=8). Significance threshold was set after Bonferroni correction. **(C)** Heat map based on the median levels of soluble cytokines/chemokines measured in supernatants obtained from patient’s PBMC (NR and R patients at different time points) after 20h stimulation with or without TLR-L (polyI:C, R848, CpGA alone or combined [mix]). Statistically significant comparisons between patient groups (inter-groups) are showed as black squares (non-parametric Kruskal-Wallis test), and the ones between T0 and another time point in a specific patient group (NR or R patient group) are illustrated as black stars (Wilcoxon matched-pairs signed rank test with Bonferroni correction). **(D-F)** Levels of cytokines/chemokines in supernatants from PBMC cultured 20h without (d; w/o stim), with mixture (e; mix: PolyI:C, R848 and CpGA) or with individual stimulation (f; PolyI:C, CpGA) in NR (triangles) and R (circles) melanoma patients at different time points of the treatment (n = 7 to 13 per group). **(A, D–F)** Bars indicate median. P-values were calculated using non-parametric Kruskal-Wallis test (straight lines) or Wilcoxon matched-pairs signed rank test with Bonferroni correction (dashed lines). Only significant statistics displayed on graphs. *P ≤ 0.05, **P ≤ 0.01.

To decipher the functionality of DC subsets in melanoma patients following anti-PD1 treatment, cytokine production (IL-12p70, IFN-λ1, IFN-α and/or TNF-α) by DC subsets after TLR stimulation was assessed in NR and R patients at several time points (T0, T3/6, T12, T24) by intracellular cytokine staining using multi-parametric flow cytometry (**Supplementary Figure 8A**). After mix stimulation (polyI:C, R848 and CpGA), higher proportions of TNF-α^+^ pDCs were observed in R compared to NR patients at T24 upon treatment (**Figure 5A**). To assess the link between PD-L1/-L2 expression by DC subsets and their functionality before anti-PD1 treatment, we performed Spearman’s correlations and found that PD-L2 expression negatively correlated with TNF-α production by circulating cDC2s in non-responders (**Figure 5B**). We also explored a large panel of cytokine/chemokine secretion by DCs after TLR stimulation. This was assessed by ProcartaPlex dosages of culture supernatants using Luminex and the median levels of each factor were illustrated in the heat map in **Figure 5C**. Without TLR stimulation, higher levels of secreted MIG and MCP-1 were found in R when compared to NR patients before or during anti-PD1 treatment (**Figure 5D**). After single or mix TLR stimulation, we observed higher levels of IL-12p70 and lower levels of TGF-β1 in R patients before and/or during immunotherapy (**Figure 5E**; **Supplementary Figure 8B**). Furthermore, after single TLR stimulation, inter-group comparisons revealed higher levels of MIP-1β and RANTES at T0, as well as higher levels of TNF-α and MIP-1α during anti-PD1 treatment in responders when compared to non-responders (**Figure 5F**). Furthermore, higher levels of IL-1β were specifically found in non-responders during immunotherapy (**Supplementary Figure 8C**). Thus, these results demonstrate that upon DC-specific stimulation higher levels of secreted IL-12p70, TNF-α and MIP-1β and lower levels of TGF-β1 were found in melanoma patients responding to anti-PD1 treatment when compared to non-responders.

### 3.8 Melanoma patients displayed distinct profiles of effector cell function and Th orientation before and during anti-PD1 therapy depending on their response to the treatment

**Figure 6:**
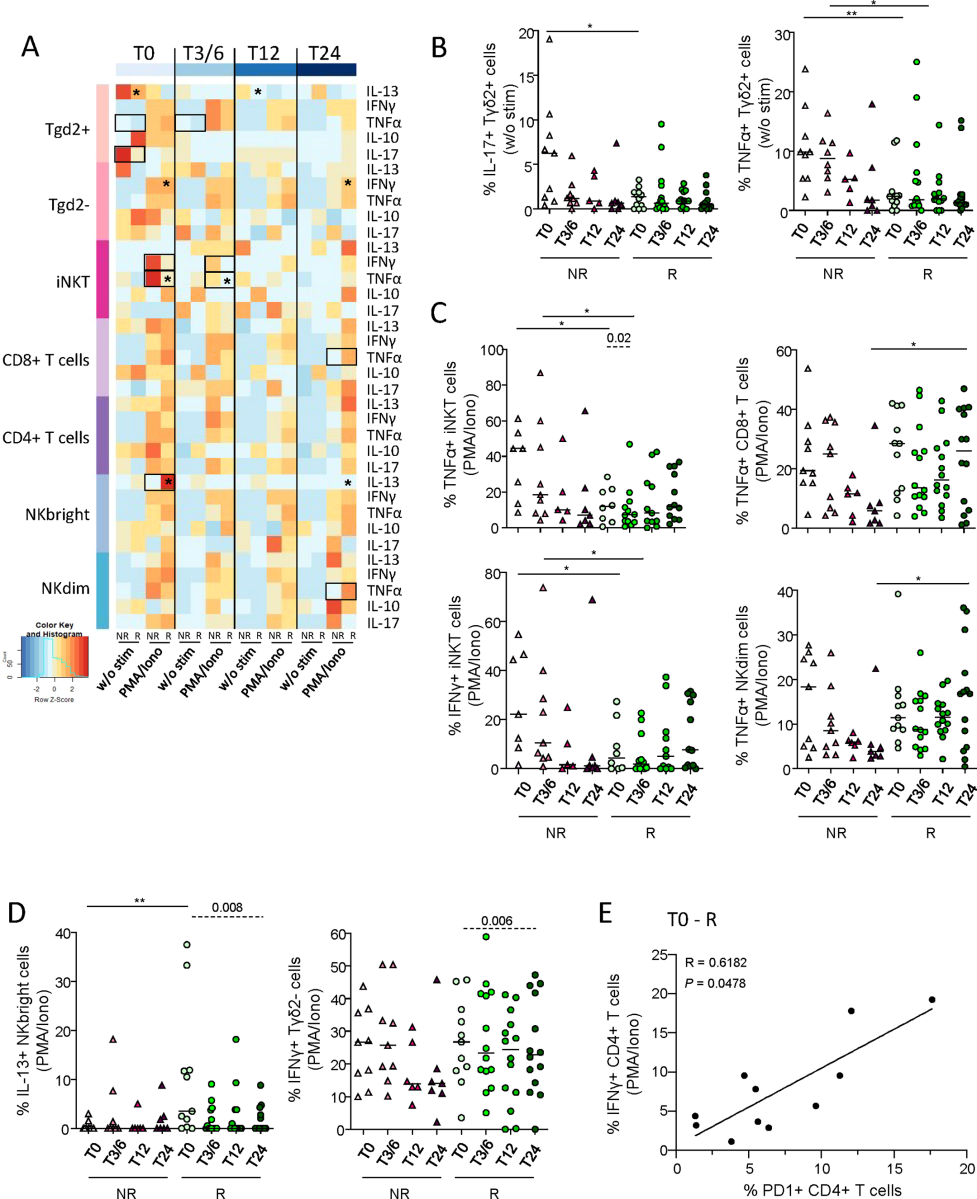
Circulating immune effector cells exhibited distinct cytokine production profiles upon stimulation depending on the clinical response of patients to anti-PD1 treatment. To inspect the functionality of circulating immune effector cells in melanoma patients following anti-PD1 treatment, cytokine production by γδ2^+^T, γδ2^-^T, iNKT, CD8^+^ T, CD4^+^ T, NK^bright^ and NK^dim^ cells was assessed by intracellular cytokine staining after PBMC stimulation by PMA/Iono, HMB-PP, IL-12/IL-18 or αGalCer using multi-parametric flow cytometry. **(A)** Heat map based on the median frequencies of cytokine-producing circulating effector cells (IL-13, IFN-γ, TNF-α, IL-10 and IL-17) upon stimulation (PMA/Iono) of patients PBMC collected at different time points of the treatment (T0, T3/6, T12, T24). Statistically significant comparisons between patient groups (inter-groups) are showed as black squares (non-parametric Kruskal-Wallis test), and the ones between T0 and another time point in a specific patient group (NR, non-responders; R, responders, intra-group) are illustrated as black stars (Wilcoxon matched-pairs signed rank test with Bonferroni correction). **(B)** Proportions of circulating IL-17^+^ or TNF-α^+^ γδ2^+^T cells after culture without any stimulation (w/o stim), derived from NR (triangles) and R (circles) melanoma patients at different time points of the treatment (n = 5 to 14 per group). **(C, D)** Following PMA/Iono stimulation, frequencies of **(C)** TNF-α-producing iNKT, CD8^+^ T or NK^dim^ cells, and IFN-γ^+^ iNKT cells, and of **(D)** IL-13-producing NK^bright^ cells and IFN-γ-producing γδ2-T cells derived from NR (triangles) and R (circles) melanoma patients at different time points of the treatment (n = 5 to 14 per group). **(E)** Spearman’s correlation between the frequency of IFN-γ^+^ CD4^+^ T cells after PMA/Iono stimulation and the proportion of PD1^+^ CD4^+^ T cells in responder melanoma patients before the start of immunotherapy (T0-R; n=11). All frequencies are indicated within the corresponding cell subset. **(B-D)** Bars indicate median. P-values were calculated using non-parametric Kruskal-Wallis test (straight lines) or Wilcoxon matched-pairs signed rank test with Bonferroni correction (dashed lines). Only significant statistics are displayed on graphs. *P ≤ 0.05, **P ≤ 0.01.

To explore the function of circulating effector cells in melanoma patients following anti-PD1 treatment, cytokine production by γδ2^+^T, γδ2^–^T, iNKT, CD8^+^ T, CD4^+^ T, NK^bright^ and NK^dim^ cells was assessed by intracellular cytokine staining after PBMC stimulation by PMA/Iono using flow cytometry (**Supplementary Figure 9A**). Heat map illustration based on cytokine production by effector cells upon PMA/Iono stimulation highlighted differences between patient groups (NR, R) before and/or during treatment (**Figure 6A**). Without any external stimulation, IL-17- and TNF-α-producing γδ2^+^T cells were more abundant in NR compared to R patients before and/or during anti-PD1 treatment (**Figure 6B**), whereas proportions of IL-13^+^ γδ2^+^T cells decreased in responders during immunotherapy (**Supplementary Figure 9B**). After PMA/Iono stimulation, higher levels of TNF-α- or IFN-γ-producing iNKT were found in NR compared to R patients before and during anti-PD1 treatment, while higher frequencies of TNF-α-secreting CD8^+^ T and NK^dim^ cells were found in responders during immunotherapy (**Figure 6C**). Higher frequencies of IL-13-producing NK^bright^ cells were also found in R when compared to NR patients before treatment (**Figure 6D**). In addition, proportions of TNF-α-producing iNKT cells, IFN-γ-producing γδ2^–^T cells and IL-13^+^ NK^bright^ cells decreased in responders during immunotherapy (**Figures 6C, D**). To study the link between PD1 expression and T-cell functionality before treatment, we performed Spearman’s correlations and found a positive correlation between PD1 expression and IFN-γ production by CD4^+^ T cells in responders (**Figure 6E**).

**Figure 7:**
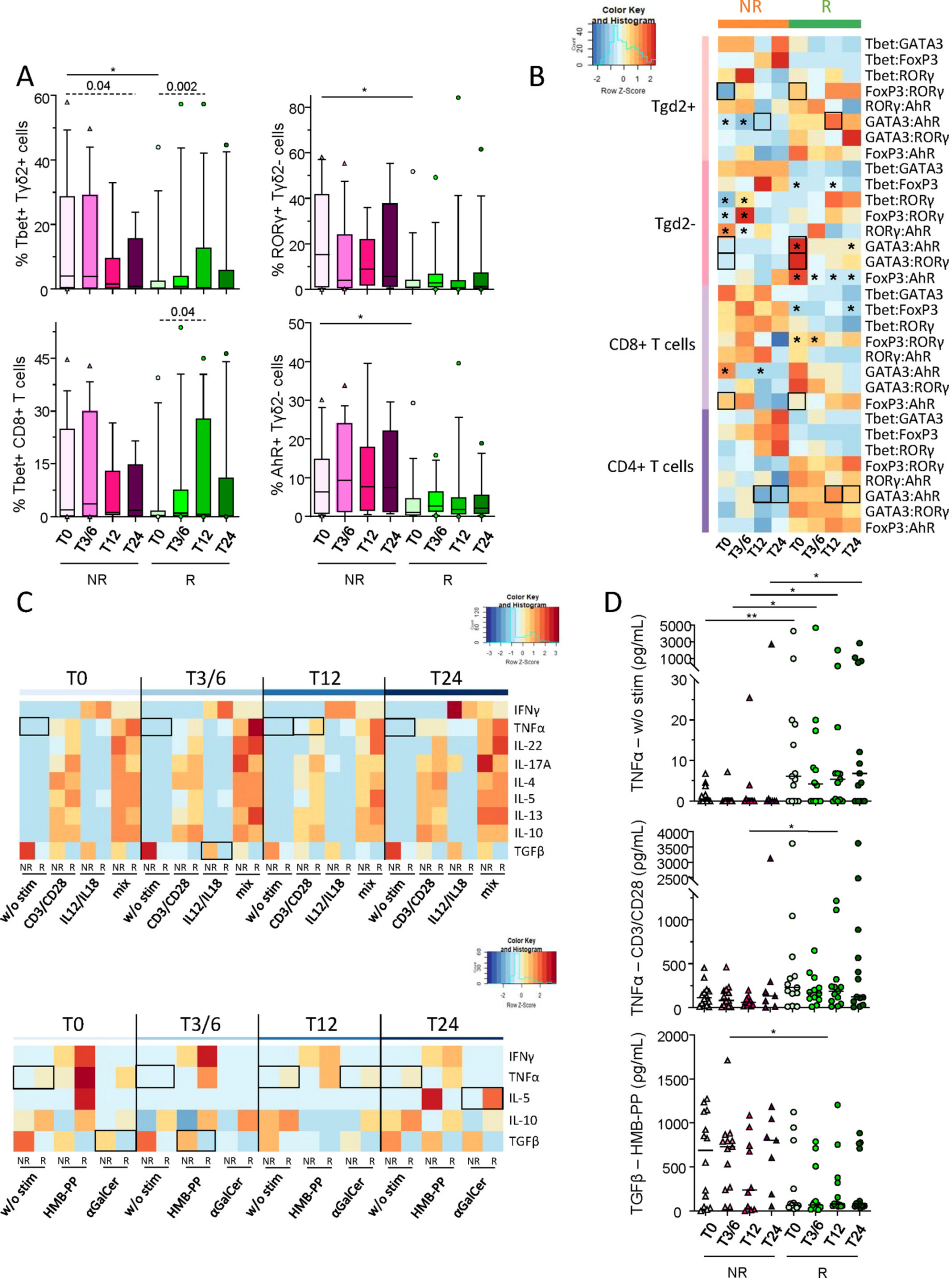
Distinct Th orientations of conventional and γδT cells, and particular cytokine secretion upon effector-specific stimulation in melanoma patients responding to anti-PD1 therapy. To decipher whether Th profile of conventional and γδT cells differed depending on the patient’s response to immunotherapy, frequencies of RORγt, AhR, Tbet, FoxP3 or GATA3-positive γδ2^+^T, γδ2^-^T, CD8^+^ T and CD4^+^ T cells were assessed in melanoma patients before and during the course of the treatment following intra-nuclear staining and flow cytometry analysis. Also, to assess the combined functionality of circulating immune effector cells in melanoma patients following anti-PD1 treatment, cytokine secretion profiles were analyzed in supernatants of PBMC after stimulation (PMA/Iono, or alone or combined CD3/CD28, HMB-PP, IL-12/IL-18 and αGalCer) by ProcartaPlex dosages using Luminex. **(A)** Frequencies of Tbet-expressing γδ2^+^T and CD8^+^ T cells, and of RORγt^+^ or AhR^+^ γδ2^-^T cells (within the corresponding cell subset) derived from non-responder (NR; triangles) and responder (R; circles) melanoma patients at different time points of the treatment (T0, T3/6, T12, T24; n = 8 to 15 per group). **(B)** Heat map based on the median ratios of the frequencies of transcription factor-expressing γδ2^+^T, γδ2^-^T, CD8^+^ T and CD4^+^ T cells derived from melanoma patients (NR, R) at different time points of the treatment. **(C)** Heat maps based on the median levels of secreted cytokines (IFN-γ, TNF-α, IL-22, IL-17A, IL-4, IL-5, IL-13, IL-10 and/or TGF-β1) found in supernatants from patients’ PBMC collected at different time points of the treatment upon culture with different stimulants alone or combined (CD3/CD28, IL-12/IL-18, HMB-PP and αGalCer). **(D)** Levels of TNF-α found after culture without any stimulation (w/o stim) or after stimulation with CD3/CD28, and of TGF-β1 after HMB-PP stimulation in PBMC supernatants derived from NR (triangles) and R (circles) melanoma patients (n = 7 to 14 per group). **(A, D)** Bars indicate median. Only significant statistics are displayed on graphs. *P ≤ 0.05, **P ≤ 0.01. **(A–D)** P-values were calculated using non-parametric Kruskal-Wallis test (straight lines on graphs or black squares on heat map) or Wilcoxon matched-pairs signed rank test (dashed lines on graphs or black stars on heat map).

We further explored Th profiles of T_conv_ and γδT cells according to patient’s response to anti-PD1 treatment through the assessment of transcription factors specific for functional orientation. Tbet, GATA3, RORγt, AhR and FoxP3 (corresponding to Th1, Th2, Th17, Th22 and Treg profiles, respectively) were studied within γδ2^+^T, γδ2^–^T, CD8^+^ T and CD4^+^ T cells before and during the course of the treatment following intra-nuclear staining and flow cytometry analysis (**Supplementary Figure 10A**). Even though Tbet-expressing γδ2^+^T cells were elevated in NR compared to R patients before anti-PD1, intra-group comparisons showed tendencies to decrease in this population during treatment in non-responders and to increase in responders, which was also the case for Tbet-expressing CD8^+^ T and CD4^+^ T cells (**Figure 7A**; **Supplementary Figure 10B**). Furthermore, non-responders displayed elevated proportions of γδ2^–^T cells with a Th17 or Th22 profile compared to responders before anti-PD1 treatment, while γδ2^–^T cells with a T_reg_ profile decreased in non-responders after the beginning of the treatment (**Figure 7A**; **Supplementary figure 10B**). In addition, by comparing transcription factor ratios, distinct Th profiles were found between NR and R patients before and/or during anti-PD1 treatment (**Figure 7B**). Before treatment, we noticed an increase of the ratios T_reg_/Th17 γδ2^+^T cells, Th2/Th22 and Th2/Th17 γδ2^–^T cells, and a decrease in T_reg_/Th22 CD8^+^ T cells in R when compared to NR patients. During the treatment, responders displayed an increase in the ratio Th2/Th22 γδ2^+^T and CD4^+^ T cells compared to non-responders (**Figure 7B**). Thus, these data pinpoint distinct Th profiles of conventional and γδ T cells in R when compared to NR melanoma patients before and/or during immunotherapy.

Lastly, to further decipher the combined functionality of circulating effector cells in melanoma patients following anti-PD1 treatment, cytokine secretion profiles were analyzed by ProcartaPlex dosages of PBMC supernatants after specific stimulation of each subset using alone or combined anti-CD3/CD28 antibodies, HMB-PP, IL-12/IL-18 and αGalCer. PCA analysis based on the median levels of secreted molecules in PBMC supernatants allowed the clustering of patients depending on their clinical response, which seemed mostly driven by TGF-β1 secretion (**Supplementary Figure 10C**). Heat map visualizations also illustrated distinct patterns of cytokine/chemokine secretion by NR and R patients upon effector-specific stimulation (**Figure 7C**). Prior to stimulation, TNF-α levels were higher in responders before and during anti-PD1 treatment (**Figure 7D**). Notably, following effector-specific stimulation, TNF-α and IL-5 secretions were higher in R compared to NR patients during anti-PD1 treatment, while TGF-β1 levels were higher in non-responders before and/or during immunotherapy (**Figure 7D**; **Supplementary Figure 10D**). Altogether, these results highlighted that anti-PD1 treatment triggers deep changes in the functional orientation of effector cells, and mostly T_conv_ and γδ T cells.

## 4 Discussion

Our study represents the first depiction of a global immunological landscape in response to anti-PD1 therapy in melanoma patients, deciphering most of circulating immune subsets together (DC subsets [cDC2, pDC, cDC1] and effectors [T, NK, γδT, iNKT cells]), as well as their extended phenotypic and functional features at a protein level. We investigated ten distinct immune cell types whose features were previously shown to be pivotal in dictating the clinical outcome of melanoma patients, and found them to be also critical in response to PD1 therapy. Our study revealed both inter-group differences according to response to treatment and modulations of patterns within each group during the course of the treatment. Our work brings answers to the current crucial medical requirements of identification of patients susceptible to respond to therapy. Before the start of the treatment (T0), we highlighted differences between NR and R patients, allowing the depiction of predictive factors of response to anti-PD1 therapy. Unsupervised analyses permitted to separate patient’s response groups according to proportions of immune cell subsets and to their phenotypic and functional features. This highlights a strong link between the immune landscape and response to or failure of anti-PD1 therapy.

The originality of our work relies on studying immune cell subsets that have not yet been explored in the context of ICB. These immune cell subsets were found to be subverted in the blood and tumor microenvironment (TME) of melanoma patients and harbor a strong impact on clinical outcomes (such as cDC1, γδ T cells). The simultaneous analysis of a large number of cell subsets allows to decipher immune cell network. Indeed, our recent work highlights that many immune cell subsets are major actors in the progression of melanoma, and unravels phenotypic modulations and functional defects of circulating and tumor-infiltrating DC subsets, T_conv_, NK, γδT and iNKT cells in melanoma patients together with dysregulation of immune cell crosstalk and networking in melanoma (10, 14, 15, 19, 33). In addition, most of the known immune parameters linked with clinical response to anti-PD1 treatment were defined at the tumor site (T-cell infiltration, proportions of Treg, MDSCs, M2-polarized tumor-associated macrophages (23, 25)) which remained difficult to assess as predictive factors in clinical routine. Among circulating immune subsets, previous studies mostly focused on T cells, or other subsets, but analyzed only proportions (27). Our work offers a unique global comprehensive analysis of immunologic landscape before and under anti-PD1 therapy, of both immune cell proportions, phenotypes and function previously shown to dictate clinical outcomes in melanoma. We unravel that all DCs and effector cell subsets are profoundly affected by anti-PD1 therapy, both phenotypically and functionally.

We observed variations of circulating immune cell proportions between patient groups before the start of the therapy and within each group during the course of the treatment, mostly driven by the proportions of cDC1s and CD8^+^ T cells. Both at baseline and/or during the course of anti-PD1 therapy, R patients were characterized by higher frequencies of cDC1s, higher frequency of CD8^+^ T cells, as well as CD8^+^/CD4^+^ T cell ratio, and lower proportion of NK^bright^ cells when compared to NR patients. Interestingly, in a melanoma mouse model, the presence of cDC1s within tumors revealed to be essential for ICB efficacy (31). In another study, Tumeh et al. found that patients responding to anti-PD1 displayed higher numbers of CD8^+^ T cells at the invasive tumor margin and inside tumors together with proliferation of intratumoral CD8^+^ T cells (29). Thus, our results reflect in the periphery changes that have already been seen at the tumor site. In addition, previous studies demonstrated associations of baseline and/or post-treatment changes in absolute numbers of lymphocytes, eosinophils, neutrophils, and monocytes with responses to ICB (23, 24, 34), further emphasizing that anti-PD1 therapy completely reshapes the immunological landscape, both in the circulation and at the tumor site.

We also revealed that the differentiation stage of effector cells represents an important feature allowing the distinction between R and NR patients. Indeed, R patients exhibited higher frequencies of circulating CM γδ2^+^T cells, while fewer frequencies in EMRA γδ2^+^T cells compared to NR patients. A same tendency was also depicted for CD8^+^ and CD4^+^ T cells, even though statistical significance was not reached. Upon anti-PD1 treatment, levels of EM γδ2^+^T cells and CM γδ2^–^T cells decreased in NR patients. Our observations are in line with a previous study in which clinical responders to anti-PD1 treatment displayed an increase in the periphery of a subset of CM CD4^+^ T cells harboring the CD27^+^Fas^–^CD45RA^–^CCR7^+^ phenotype (35). In addition, circulating baseline levels of CD8^+^CD45RO^+^ EM T cells correlated with the clinical response of melanoma patients to ipilimumab (36). Tumors of patients who respond to anti-PD1 therapy displayed higher baseline EOMES^+^CD69^+^CD45RO^+^ EM T cells (that were also Tbet^hi^) (37). In a preclinical mouse model of melanoma, anti-PD1 immunotherapy triggered changes in both tumor-infiltrating and circulating T cell subsets, with a marked increase in EM and CM CD8^+^ T cells associated with durable responses (38). Altogether, these studies support a predictive role of memory (CM/EM) T_conv_ and γδ2^+^ T cells.

Our study highlights the importance of DC and effector cell subsets’ features in the response to anti-PD1, by underlining a distinct remodeling of ICP expression profile, activation status and NCR patterns of circulating immune subsets both between response group and within each group during the duration of the treatment. Before the start of the treatment, R patients displayed higher frequencies of PD1-expressing CD4^+^ T cells (and potentially γδ2^+^T cells), and endured significant PD1 downregulation on all effectors during the course of therapy. In line with our study, an immune profiling performed on fresh metastatic melanoma samples prior to anti-PD1 therapy revealed that an increase of fractions of tumor-infiltrating (partially exhausted) PD1^hi^CTLA4^hi^CD8^+^ T cells strongly correlated with response to therapy (39), sustaining PD1 expression on T cells as a reliable marker of response to treatment in both the periphery and the tumor. Interestingly, anti-PD1 therapy differentially affects ICP expression profile of DCs from R and NR patients. During the course of the treatment, we observed specifically in the NR group an increase in the frequencies of PD-L2-expressing cDC1s, cDC2s and pDCs, TIM3-expressing cDC1s and pDCs, and CD70-expressing cDC1s together with a decreased frequency of ICOS-L^+^ cDC2s at T3/6, T12 and/or T24 compared to baseline. Responders displayed decreased proportions of LAG3- or CD40-expressing pDCs during treatment at T24 compared to baseline. ICP expression profile on effectors also fluctuated depending on patients’ clinical response. At T0, R patients displayed higher proportions of 41BB-expressing γδ2^+^T and TIGIT-expressing γδ2^–^T cells, and lower proportions of GITR^+^ γδ2^–^T cells in comparison to NR patients. During the course of anti-PD1 therapy, higher proportions of TIGIT- and/or LAG3-expressing γδ2^–^T and iNKT cells associated with decreased frequencies of GITR-expressing γδ2^–^T and iNKT, 41BB^+^ NK^bright^ and CD27^+^ NK^dim^ cells were observed in R patients, while NR patients displayed decreased proportions of TIGIT^+^ NK^dim^ and CD27^+^ NK^bright^ cells, concomitantly to increased frequencies of ICOS-expressing NK and γδT cells. Such remodelling of ICP expression profile on effector cells by anti-PD1 has been observed in other studies both in the circulation and at the tumor site. Among many variables analyzed in blood, higher levels of PD1^+^ CD4^+^ T cells, detectable CD137 (41BB) expression on circulating CD8^+^ T cells, as well as lower PD-L1 expression on circulating CD4^+^ or CD8^+^ T cells were the best predictors associated with the likelihood to respond to anti-PD1/-CTLA4 treatment (34). Tumors from patients not responding to anti-PD1 therapy were enriched in T cells expressing alternative ICPs, such as ICOS and TIGIT (37). In addition, we found interesting correlations between ICP expression on the same cell subsets, such as negative correlation between PD1 and TIGIT on circulating CD8^+^ T cells in R patients or positive correlation between PD1 and GITR on circulating γδ2^–^T cells in NR patients. Taken together, these results strongly suggest that many ICPs are actually crucial for the response to anti-PD1 therapy and are cross-regulated, thus paving the way to design therapies combining both inhibitory and agonistic antibodies to overcome resistance to anti-PD1 therapy in non-responders.

Interestingly, anti-PD1 also modulated the activation status and NCR patterns of effector cells. Both before and upon treatment, responders exhibited lower proportions of CD69-, CD86- and/or CD40-expressing γδ2^–^T, γδ2^+^T and iNKT cells compared to non-responders. During the course of treatment, a reduction in the frequencies of NKG2D-expressing γδ2^+^T cells and in the expression level of NKG2D on γδ2^–^T and γδ2^+^T cells were observed in R patients, concomitantly to reduced proportions of NKp46^+^ NK^dim^ and NK^bright^ cells together with lower NKG2A levels on NK^bright^ cells compared to non-responders. These observations sustain that anti-PD1 also affects the ability of NK and γδT cells to exhibit their cytotoxic potential through modulation of their activation status and ability to recognize and lyse tumor cells.

We further highlighted that anti-PD1 treatment modulates functionality of DC subsets and triggers deep changes in the functional orientation of T_conv_ and γδT cells. Indeed, patients responding to immunotherapy exhibited before treatment distinct cytokine/chemokine production and secretion profiles upon DC- or effector-specific stimulation compared to non-responders. Without any external stimulation, R patients displayed higher levels of secreted TNF-α, MIG and MCP-1 from PBMC compared to NR patients. In contrast, NR patients displayed higher proportions of IL-17- and TNF-α-producing γδ2^+^T cells and elevated proportions of Th17/Th22-oriented γδ2^–^T cells compared to R patients. After TLR stimulation, R patients displayed higher levels of IL-12p70, MIP-1β and RANTES, together with lower levels of TGF-β1 compared to NR patients. After effector-specific stimulation, R patients displayed lower levels of IFN-γ- and TNF-α-producing iNKT cells, higher frequencies of IL-13-producing NK^bright^ cells, as well as lower TGF-β1 levels compared to NR. Such different functional orientation between DCs and effectors was also depicted during the course of anti-PD1 therapy. After TLR stimulation, higher proportions of TNF-α^+^ pDCs as well as higher levels of IL-12p70, TNF-α and MIP1-α, and lower levels of TGF-β1 were observed in R patients compared to NR patients. After PMA/Iono stimulation, higher levels of IFN-γ- and TNFα-producing iNKT cells were found in NR patients, while higher frequencies of TNF-α-secreting CD8^+^ T and NK^dim^ cells were found in R patients. Following effector-specific stimulation, TGF-β1 levels were higher in NR patients while TNF-α and IL-5 secretions were higher in R patients. Notably, an increase in Tbet-expressing γδ2^+^T cells, CD8^+^ T and CD4^+^ T cells occurred in responders during the course of treatment, signing a Th1 orientation of the effectors driven by anti-PD1 therapy. The importance of an active IFN-γ pathway in the clinical efficacy of anti-PD1 treatment concords with other studies in the field. Analysis of melanoma tumor biopsies before treatment by anti-PD1 therapy identifies a T-cell inflamed TME –characterized by an active IFN-γ related profile, cytotoxic effector molecules and cytokines– as positively associated with a clinical benefit (40). During ICB therapy, another study revealed that increased T cell infiltration and downstream IFN-γ signalling signatures drive the clinical response of melanoma patients (41).

Soluble factors found in the plasma of melanoma patients do not clearly allow the distinction of responders from non-responders before the beginning of anti-PD1 treatment. However, we observed an increase in soluble CD73 (sCD73) in R patients compared to NR patients at T24. sCD73 participates in extracellular production of adenosine that downregulates inflammatory and immune responses. Interestingly, sCD73 has been previously highlighted as a potential prognostic and predictive marker of treatment response. sCD73 enzymatic activity was found to be associated with poor clinical outcome in patients with metastatic melanoma, and elevated basal levels of sCD73 enzymatic activity were associated with lower response rates to anti-PD1 therapy (42). Besides, during anti-PD1 treatment, we observed that R patients exhibited an increase of soluble CD27, PD-L2 and perforin, and a decrease of sGITR. Zhou et al. previously reported that, despite elevated levels of sPD-L1 in progressive patients before treatment, patients showing an increase of circulating sPD-L1 after five months of PD1 blockade had greater likelihood of developing a partial response (22). Besides, exploration of cytokine dynamics revealed higher pre-treatment serum levels of IL-8, IL-6, IFN-γ, IL-10 and TGF-β to be associated with response to therapy (43, 44).

Altogether, our work revealed that phenotypic and functional features of DC subsets (cDC2s, pDCs, cDC1s) and effectors (T, NK, γδT, iNKT cells) are promising pathways to explore in the context of ICB. The present comprehensive circulating immune profiling is consistent with earlier studies, yet unravelling many other cell subsets together with phenotypic and functional features involved in the response or resistance to anti-PD1 therapy. Our study emphasizes the major role played by cDC1s, T_conv_ and γδ2^+^T cells in the response to anti-PD1 therapy. Integration of the phenotypic and functional features together with clinical parameters ultimately provided a unique comprehensive understanding of the biologic impact of anti-PD1 therapy on the crucial immune cell subsets participating in anti-tumor responses. Such exploration participates in depicting predictive biomarkers and signatures of response or non-response to anti-PD1 treatment, allowing to early evaluate the clinical benefit of treatment, and to uncover mechanisms of response or non-response to the treatment, thus better understanding the mode of action of ICB. We highlighted immunometrics promising for future prospective validation, which will contribute in optimizing the therapeutic efficacy of anti-PD1 treatment, better orienting therapeutic choices and in designing pertinent combination strategies to improve NR patient clinical benefits in the future. These strategies can target other ICP, or induce the reprograming of DC functions to restore potent anti-tumor effectors, or can exploit the potential of γδT cells. Indeed, our study, based on simultaneous analyses of multiple immune cell subsets, crucially permitted to decipher immune cell networking in response to anti-PD1 therapy, allowing anticipating pertinent combotherapies. In addition, our study focuses on cells that are still underexplored in the context of ICB (i.e., cDC1, cDC2, pDC, γδT cells), but which have themselves a high potential for exploitation as a target or vector for immunotherapy. Our study brings also pertinent information for the next generation of ICP receptors being harnessed in the clinic especially in melanoma, such as LAG3, TIM3 and TIGIT (45, 46). We described the expression of these ICPs on both DC subsets and/or effector cells, and their modulation under anti-PD1 therapy, offering strong rational to design pertinent combination strategies. Better understanding the ICP-mediated crosstalk between tumor and immune cells as well as between DCs and effector cells is essential to design new strategies that are more effective and maximize clinical success.

Overall, our results provide insights into the longitudinal immunological landscape sustaining favourable clinical responses or resistance to first-line anti-PD1 therapy in melanoma patients. Our work opens promising avenue for enhancing the efficacy of immunotherapy against multiple tumors, and achieving better clinical successes in the future.

## Data Availability

The original contributions presented in the study are included in the article/**Supplementary Material**. Further inquiries can be addressed to the corresponding author.
