# Supplementary material for: Circulating immune landscape in melanoma patients undergoing anti-PD1 therapy reveals key immune features according to clinical response to treatment
Source: Front Immunol. 2024 Dec 2;15:1507938. doi: 10.3389/fimmu.2024.1507938 (PMC11646980; doi:10.3389/fimmu.2024.1507938)
Supplement: Supplementary file 1 [file DataSheet1.pdf]

Supplementary Material

1 Supplementary Materials and Methods: Key resources table

REAGENT or RESOURCE	SOURCE	IDENTIFIER
**Antibodies**		
Anti-human CD45 monoclonal antibody (Mouse, Clone HI30), Brilliant Violet 570™ conjugated	Biolegend	Cat # 304034; RRID: AB_2563426
Anti-human Lineage Cocktail (CD3, CD14, CD16, CD19, D20, CD56) (Mouse; Clones OKT3, M5E2, 3GB, HIB19, 2H7, HCD56), Brilliant Violet 510™ conjugated	Biolegend	Cat # 348807; RRIDs: AB_2561376, AB_2561379, AB_2561380, AB_2561381, AB_2561721, AB_2561385
Anti-human HLA-DR monoclonal antibody (Mouse, Clone L243), APC-H7 conjugated	BD Biosciences	Cat # 641411; RRID: AB_2870307
Anti-human CD11c monoclonal antibody (Mouse, Clone B-ly6), PerCP-Cy5.5 conjugated	BD Pharmingen	Cat # 565227; RRID: AB_2739122
Anti-human CD1c monoclonal antibody (Mouse, Clone L161), PE-Cy7 conjugated	Biolegend	Cat # 331516; RRID: AB_2275574
Anti-human CD303 (BDCA-2) monoclonal antibody (Mouse, Clone AC144), APC conjugated	Miltenyi Biotec	Cat # 130-113-190; RRID: AB_2726015
Anti-human CD141 (BDCA-3) monoclonal antibody (Mouse, Clone AD5-14H12), APC conjugated	Miltenyi Biotec	Cat # 130-113-314; RRID: AB_2733313
Anti-human Vδ2 TCR monoclonal antibody (Mouse, Clone B6), FITC conjugated	BD Pharmingen	Cat # 555738; RRID: AB_396081
Anti-human TCR Vα24-Jα18 (iNKT cell) monoclonal antibody (Mouse, Clone 6B11), PerCP-Cy5.5 conjugated	Biolegend	Cat # 342914; RRID: AB_2562455
Anti-human TCR Vα24-Jα18 (iNKT cell) monoclonal antibody (Mouse, Clone 6B11), PE-Cy7 conjugated	Biolegend	Cat # 342912; RRID: AB_2562230
Anti-human CD3 monoclonal antibody (Mouse, Clone SK7), APC-H7 conjugated	BD Pharmingen	Cat # 560176; RRID: AB_1645475
Anti-human TCRγδ monoclonal antibody (Mouse, Clone B1), Brilliant Violet 421™ conjugated	BD Horizon	Cat # 562560; RRID: AB_2737655
Anti-human CD56 monoclonal antibody (Mouse, Clone NCAM16.2), Brilliant Violet 510™ conjugated	BD Horizon	Cat # 563041; RRID: AB_2732786
Anti-human CD8 monoclonal antibody (Mouse, Clone RPA-T8), Brilliant Violet 786™ conjugated	BD Horizon	Cat # 563823; RRID: AB_2687487
Anti-human CD80 monoclonal antibody (Mouse, Clone MAB104), FITC conjugated	Beckman Coulter	Cat # IM1853U; RRID: AB_131376
Anti-human CD40 monoclonal antibody (Mouse, Clone MAB89), PE conjugated	Beckman Coulter	Cat # IM1936U; RRID: AB_131687

Anti-human CD86 monoclonal antibody (Mouse, Clone 2331), Alexa Fluor® 700 conjugated	BD Pharmingen	Cat # 561124; RRID: AB_10564087
Anti-human CD69 monoclonal antibody (Mouse, Clone FN50), PE-Cy5 conjugated	BD Pharmingen	Cat # 555532; RRID: AB_395917
eBioscience™ anti-human CD25 monoclonal antibody (Mouse, Clone BC96), APC conjugated	Invitrogen	Cat # 17-0259-42; RRID: AB_1582219
Anti-human CD274 (PD-L1) monoclonal antibody (Mouse, Clone MIH1), Brilliant Violet 421™ conjugated	BD Horizon	Cat # 563738; RRID: AB_2738396
Anti-human CD273 (PD-L2) monoclonal antibody (Mouse, Clone MIH18), Brilliant Violet 650™ conjugated	BD Horizon	Cat # 563844; RRID: AB_2738447
Anti-human 41BB-Ligand/TNFSF9 monoclonal antibody (Mouse, Clone #282220), Alexa Fluor® 488 conjugated	Bio-Techne	Cat # FAB2295G-100UG; RRID: AB_2207515
eBioscience™ anti-human CD275 (B7-H2) monoclonal antibody (Mouse, Clone MIH12), PE conjugated	Invitrogen	Cat # 12-5889-42; RRID: AB_10853668
Anti-human GITR-Ligand/TNFSF18 monoclonal antibody (Mouse, Clone #109101), Alexa Fluor® 700 conjugated	Bio-Techne	Cat # FAB6941N; RRID: AB_2207259
Anti-human OX40-Ligand (CD252) monoclonal antibody (Mouse, Clone ik-1), Brilliant Violet 421™ conjugated	BD Horizon	Cat # 563766; RRID: AB_2738412
Anti-human CD70 monoclonal antibody (Mouse, Clone Ki-24), Brilliant Violet 786™ conjugated	BD Horizon	Cat # 565338; RRID: AB_2739192
Anti-human LAG-3 (CD223) monoclonal antibody (Mouse, Clone T47-530), Brilliant Violet 786™ conjugated	BD OptiBuild	Cat # 744727; RRID: AB_2742438
Anti-human TIM-3 (CD366) monoclonal antibody (Mouse, Clone 7D3), Brilliant Violet 650™ conjugated	BD Horizon	Cat # 565564; RRID: AB_2722547
eBioscience™ anti-human CD357 (AITR/GITR) monoclonal antibody (Mouse, Clone eBioAITR), PE-Cy7 conjugated	Invitrogen	Cat # 25-5875-42; RRID: AB_2573485
Anti-human CD279 (PD-1) monoclonal antibody (Mouse, Clone MIH4), Brilliant Violet 650™ conjugated	BD Horizon	Cat # 564324; RRID: AB_2738746
eBioscience™ anti-human CD366 (TIM3) monoclonal antibody (Mouse, F38-2E2), PE conjugated	Invitrogen	Cat # 12-3109-42; RRID: AB_2572605
Anti-human CD137 monoclonal antibody (Mouse, Clone 4B4-1), PE-Cy5 conjugated	BD Pharmingen	Cat # 551137; RRID: AB_394067
eBioscience™ anti-human CD152 (CTLA-4) monoclonal antibody (Mouse, Clone 14D3), PE-Cy7 conjugated	Invitrogen	Cat # 25-1529-42; RRID: AB_2573406

eBioscience™ anti-human CD223 (LAG-3) monoclonal antibody (Mouse, Clone 3DS223H), APC conjugated	Invitrogen	Cat # 17-2239-42; RRID: AB_2573186
Anti-human CD278 monoclonal antibody (Mouse, Clone DX29), Brilliant Violet 650™ conjugated	BD Horizon	Cat # 563832; RRID: AB_2738439
Anti-human CD134 monoclonal antibody (Mouse, Clone L106), Brilliant Violet 786™ conjugated	BD OptiBuild	Cat # 744746; RRID: AB_2742454
eBioscience™ anti-human CD336 (NKp44) monoclonal antibody (Mouse, Clone 44.189), PE conjugated	Invitrogen	Cat # 12-3369-42; RRID: AB_2572607
Anti-human CD314 (NKG2D) monoclonal antibody (Mouse, Clone 1D11), PE-Cy7 conjugated	BD Pharmingen	Cat # 562365; RRID: AB_11153309
Anti-human CD335 (NKp46) monoclonal antibody (Mouse, Clone 9E2/NKp46), APC conjugated	BD Pharmingen	Cat # 558051; RRID: AB_398653
Anti-human NKG2C/CD159c monoclonal antibody (Mouse, Clone # 134591), Alexa Fluor® 700 conjugated	Bio-Techne	Cat # FAB138N-100; RRID: AB_2132982
Anti-human CD337 (NKp30) monoclonal antibody (Mouse, Clone p30-15), Brilliant Violet 650™ conjugated	BD OptiBuild	Cat # 743171; RRID: AB_2741322
Anti-human NKG2A (CD159a) monoclonal antibody (Mouse, Clone 131411), Brilliant Violet 786™ conjugated	BD OptiBuild	Cat # 747917; RRID: AB_2872378
Anti-human CD27 monoclonal antibody (Mouse, Clone M-T271), PE conjugated	BD Pharmingen	Cat # 555441; RRID: AB_395834
Anti-human CD45RA monoclonal antibody (Mouse, Clone HI100), PE-Cy7 conjugated	BD Pharmingen	Cat # 560675; RRID: AB_1727498
eBioscience™ anti-human TIGIT monoclonal antibody (Mouse, Clone MBSA43), Alexa Fluor™ 700 conjugated	Invitrogen	Cat # 56-9500-42; RRID: AB_2815260
eBioscience™ anti-human ROR gamma (t) monoclonal antibody (Mouse, Clone AFKJS-9), PE conjugated	Invitrogen	Cat # 12-6988-82; RRID: AB_1834470
eBioscience™ anti-human GATA-3 monoclonal antibody (Mouse, Clone TWAJ), PE-Cy5 conjugated	Invitrogen	Cat # 15-9966-42; RRID: AB_2811756
eBioscience™ anti-human AHR monoclonal antibody (Mouse, Clone FF3399), PE-Cy7 conjugated	Invitrogen	Cat # 25-9854-42; RRID: AB_2573556
eBioscience™ anti-human FOXP3 monoclonal antibody (Mouse, Clone PCH101), Alexa Fluor® 700 conjugated	Invitrogen	Cat # 56-4776-41; RRID: AB_1582210
Anti-human T-bet monoclonal antibody (Mouse, Clone O4-46), Brilliant Violet 650™ conjugated	BD Horizon	Cat # 564142; RRID: AB_2738616
Anti-human IL-29/IFN-lambda 1 monoclonal antibody (Mouse, Clone 247801), unconjugated	R&D Systems	Cat # MAB-15981-100; RRID: AB_2125340
Anti-human IFN-α monoclonal antibody (Mouse, Clone LT27:295), PE conjugated	Miltenyi Biotec	Cat # 130-123-708; RRID: AB_2889689

Anti-human TNF monoclonal antibody (Mouse, Clone MAb11), Alexa Fluor® 700 conjugated	BD Pharmingen	Cat # 557996; RRID: AB_396978
Anti-human IL-12 (p40/p70) monoclonal antibody (Mouse, Clone C8.6), Brilliant Violet 421™ conjugated	BD Horizon	Cat # 565023; RRID: AB_2739045
Anti-human IL-13 monoclonal antibody (Rat, Clone JES10-5A2), PE conjugated	BD Pharmingen	Cat # 559328; RRID: AB_397225
Anti-human IL-17A monoclonal antibody (Mouse, Clone N49-653), PerCP-Cy5.5 conjugated	BD Pharmingen	Cat # 560799; RRID: AB_2033981
Anti-human IFN-γ monoclonal antibody (Mouse, Clone 25723.11), APC conjugated	BD FastImmune	Cat # 341117; RRID: AB_2264629
Anti-human IL-10 monoclonal antibody (Rat, Clone JES3-9D7), Brilliant Violet 650™ conjugated	BD Horizon	Cat # 564051; RRID: AB_2738565
**Biological samples**		
Patient blood from [#2023-A01722-43]	Provided by CHU Grenoble Alpes	N/A
**Chemicals, peptides, and recombinant proteins**		
LIVE/DEAD™ Fixable Red Dead Cell Stain Kit, for 488 nm excitation	Invitrogen	Cat # L-34972
Mix-n-Stain™ CF® Dye Antibody Labeling Kit, dye CF®488A	Biotium	Cat # 92233
Brilliant Stain Buffer (RUO)	BD Horizon	Cat # 566349
Polyinosine-polycytidylic acid, Poly(I :C) (HMW)	Invivogen	Cat # tlrl-pic
Resiquimod (R848), Imidazoquinoline compound	Invivogen	Cat # tlrl-r848
Class A CpG oligonucleotide ODN 2336 (CpG_A)	Invivogen	Cat # tlrl-2336-1
Phorbol 12-myristate 13-acetate (PMA)	Sigma-Aldrich	Cat # P-8139
Ionomycine calcium salt from *Streptomyces conglobatus*	Sigma-Aldrich	Cat # I0634
(E)-1-Hydroxy-2-methyl-2-butenyl 4-pyrophosphate lithium salt (HMBPP)	Sigma-Aldrich	Cat # 95098
ImmunoCult™ human CD3/CD28 T cell activator	StemCell	Cat # 10971
Recombinant Human IL-12 Protein, unconjugated	Bio-Techne	Cat # 219-IL-025
Recombinant Human IL-18/IL-1F4 Protein, unconjugated	Bio-Techne	Cat # B003-5
alpha-Galactosylceramide, NKT cell stimulator	Abcam	Cat # ab144262
PeproTech® Human IL-2 recombinant protein, unconjugated	Gibco	Cat # 200-02
RPMI 1640 Medium, GlutaMAX™ Supplement, HEPES	Gibco	Cat # 72400-054
Gentamicin (10 mg/mL)	Gibco	Cat # 15710-049
Sodium pyruvate solution	Sigma-Aldrich	Cat # S8636
MEM Non-Essential Amino Acids Solution (100X)	Gibco	Cat # 11140-035
Fetal Bovine Serum, qualified, One Shot™ format, Brazil	Gibco	Cat # A3160801
Dimethylsulfoxyde (DMSO)	Sigma	Cat # D2650
FACS™ lysing solution 10X concentrate	BD Biosciences	Cat # 349202

**Critical commercial assays**		
Cytofix/Cytoperm™ Plus Fixation/Permeabilization Solution Kit with BD GolgiPlug™ (RUO)	BD Biosciences	Cat # 555028 ; RRID : AB_2869013
eBioscience™ FoxP3 / Transcription Factor Staining Buffer Set	Invitrogen	Cat # 00-5523-00
Human ProcartaPlex™ Mix&Match 17-plex	Invitrogen	Cat # PPX-17-MX2XANC
Human ProcartaPlex™ Mix&Match 4-plex	Invitrogen	Cat # PPX-04-MX47XT6
Human ProcartaPlex™ Mix&Match 8-plex	Invitrogen	Cat # PPX-08-MX32379
TGF-beta 1 Human ProcartaPlex™ Simplex Kit	Invitrogen	Cat # EPX01A-10249-901
ProcartaPlex™ Human Immune Checkpoint Panel, 37plex	Invitrogen	Cat # EPX370-15846-901
**Software and algorithms**		
GraphPad Prism 9.3.1	GraphPad Software	N/A
FACSDiva™ 9.0.1	BD Biosciences	N/A
xPONENT® software	Bio-Rad	N/A
RStudio 4.3.2	R	N/A

2 Supplementary Figures and Tables

2.1 Supplementary Figures

Supplementary Figure 1

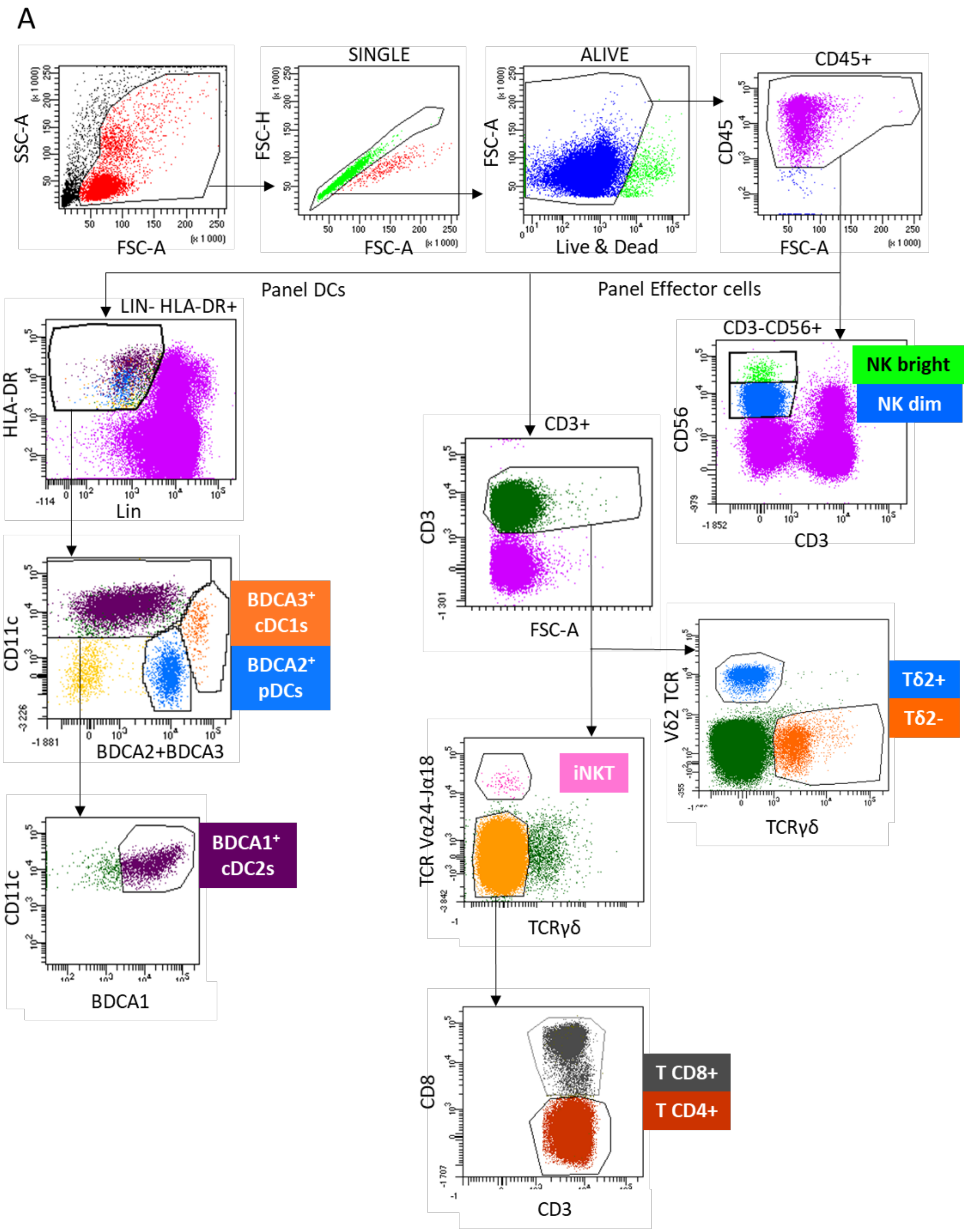

Supplementary Figure 1

B

	FITC	APC-H7	BV421
Tube 1	-	CD3	pan TCRγδ
Tube 2	TCRδ2	CD3	-
Tube 3	TCRδ2	CD3	pan TCRγδ

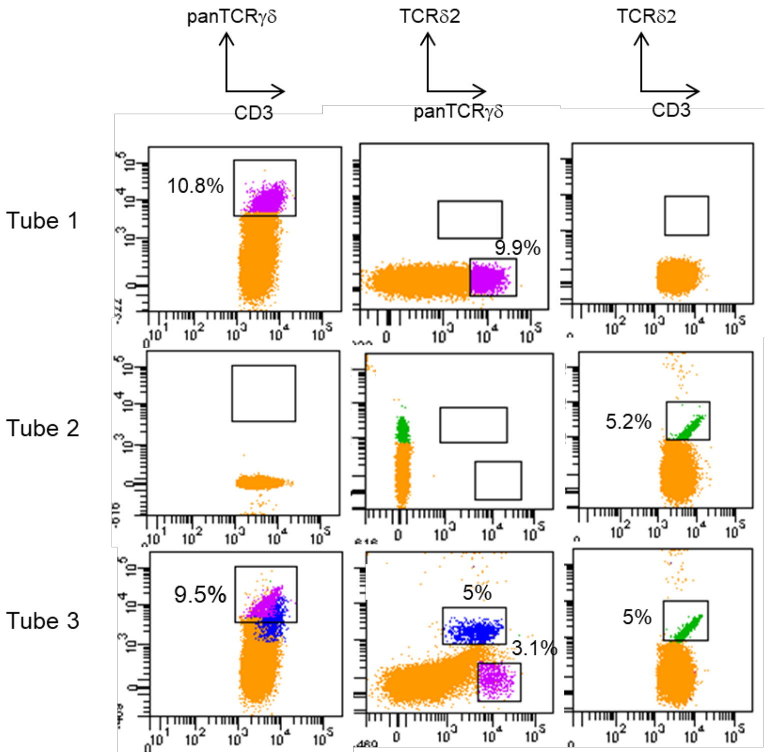

Supplementary Figure 1. (a) Gating strategy allowing to simultaneously depict DC subsets (cDC1s, cDC2s and pDCs) or effector cells ($\gamma\delta2^{+}$T, $\gamma\delta2^{-}$T, iNKT, $CD4^{+}$ T, $CD8^{+}$ T, NK^{bright} and NK^{dim}) on blood samples derived from melanoma patients. This multi-parametric flow cytometry approach allows to study the three major human DC subsets and seven effector cell populations in the circulation by using specific surface markers. FSC-A, FSC-H and SSC-A allowed the elimination of cell debris and doublets, while Live&Dead cell staining excluded dead cells. Within $CD45^{+}$ cells, different cell markers were used to obtain DC subsets and effector immune cells. On one hand, the three major DC subsets were pinpointed from $Lin^{-}HLA\text{-}DR^{+}$ cells by using CD11c, BDCA1, BDCA2 and BDCA3 markers as follows: $CD11c^{dim}BDCA3^{+}$ cDC1s, $CD11c^{+}BDCA1^{+}$ cDC2s and $CD11c^{-}BDCA2^{+}$ pDCs. On the other hand, NK cells were defined as $CD45^{+}CD3^{-}CD56^{+}$ cells (including $CD56^{dim}$ and $CD56^{bright}$ NK subsets) and all other effector cells were derived from $CD45^{+}CD3^{+}$ cells using the following markers: TCRγδ, Vδ2TCR, TCRVα24-Jα18 and CD8. Thus, $\gamma\delta2^{+}$T cells were defined as $V\delta2TCR^{+}$ cells, $\gamma\delta2^{-}$T cells were demarcated as $TCR\gamma\delta^{+}V\delta2TCR^{-}$ cells, iNKT were depicted as $TCR\gamma\delta^{-}TCRV\alpha24\text{-}J\alpha18^{+}$ cells, $CD8^{+}$ T cells were highlighted as $TCR\gamma\delta^{-}TCRV\alpha24\text{-}J\alpha18^{-}CD8^{+}$ cells and $CD4^{+}$ T cells were determined as $TCR\gamma\delta^{-}TCRV\alpha24\text{-}J\alpha18^{-}CD8^{-}$ cells. (b) Gating of γδT cells and its subsets by flow cytometry. CD3, panTCRγδ and TCRδ2 antibodies were used together (tube 3) or in combination of two to perform FMO controls (tubes 1 and 2). Dotplots depicting whole γδT cells (CD3+ panTCRγδ+), Tδ2+ cells (panTCRγδlow/neg TCRδ2+ or CD3+ TCRδ2+), and Tδ2- cells (panTCRγδ positive TCRδ2-).

Supplementary Figure 2

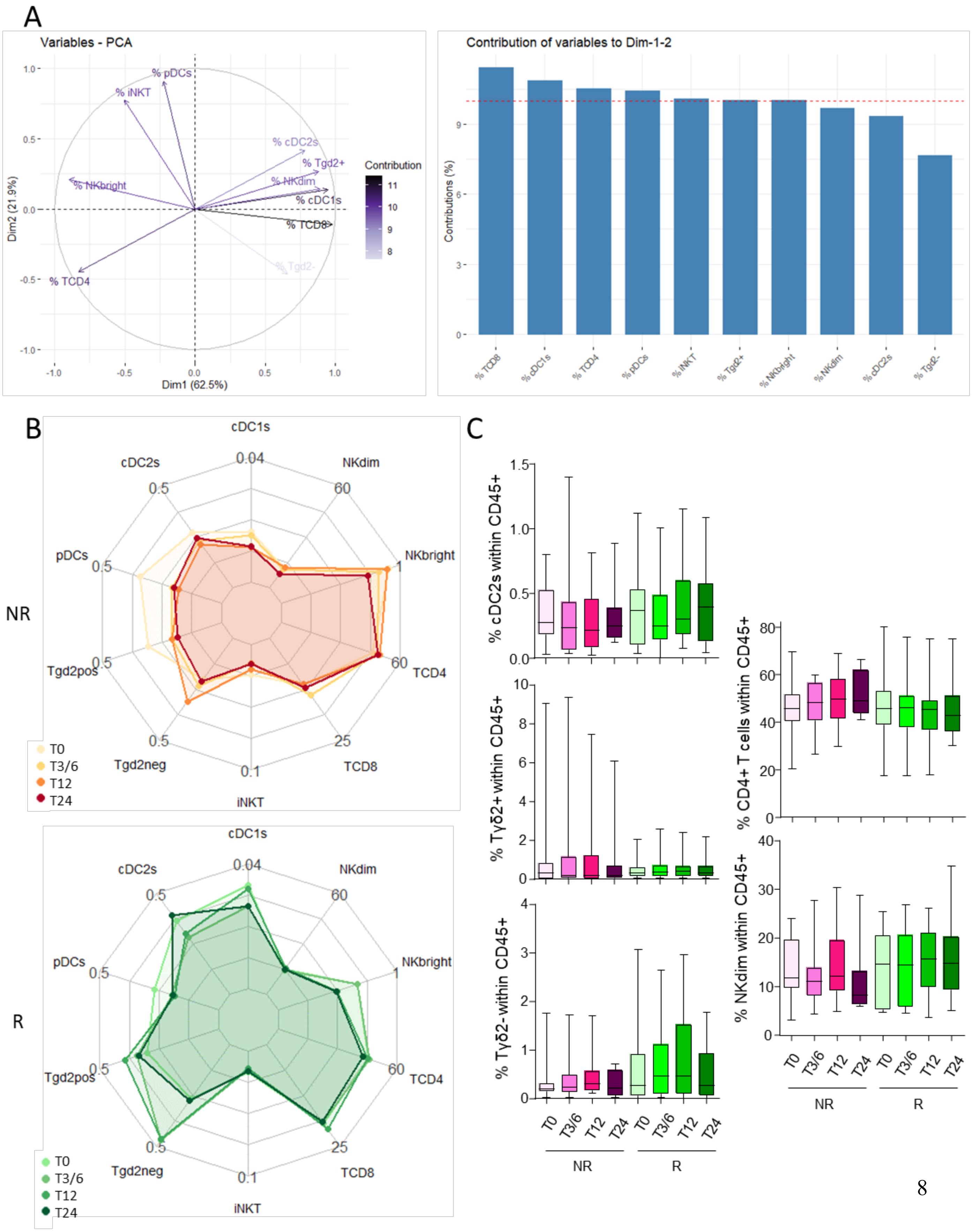

Supplementary Figure 2

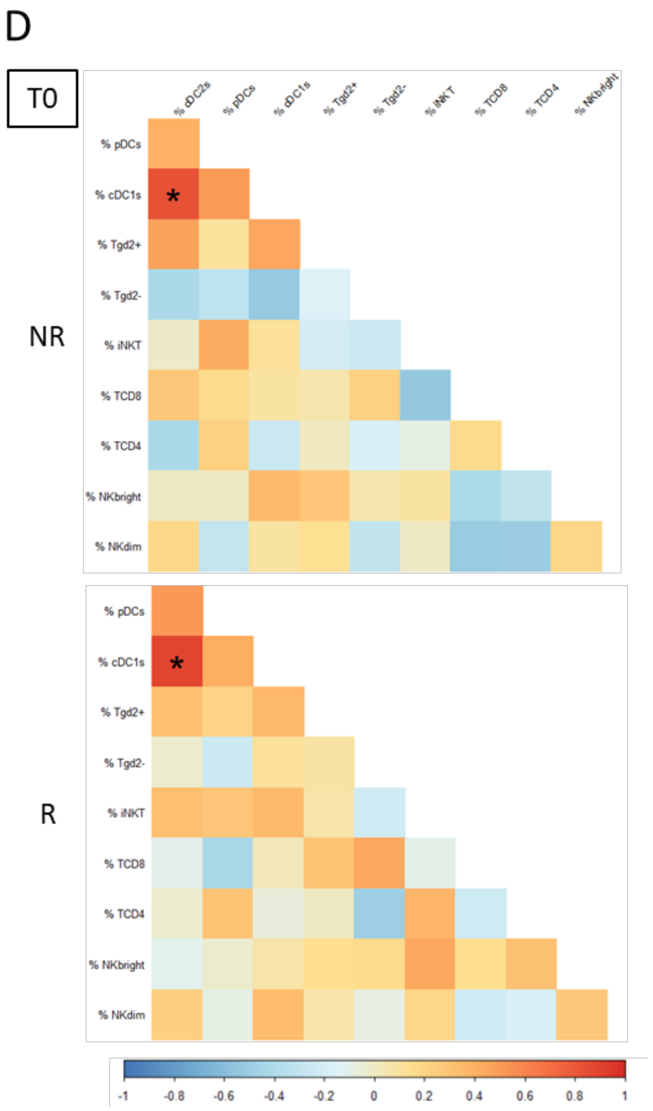

Supplementary Figure 2. Different frequencies of circulating immune cell subpopulations in melanoma patients according to the clinical response to anti-PD1 treatment. The frequencies of ten immune cell subpopulations were evaluated in the circulation of melanoma patients treated with anti-PD1 using multi-parametric flow cytometry, and compared depending on the patients' clinical response to the treatment. (a) Graph of variables (left panel) and contribution of variables to PC1 and PC2 (right panel) relative to the PCA analysis shown on Figure 1b. (b) Radar plots showing the median frequencies of the immune cell subpopulations studied at different time points of the treatment in NR (top panel) or R (bottom panel) melanoma patients. (c) Box and whiskers plots illustrating the frequencies of cDC2s, $\gamma\delta2^{+}$T, $\gamma\delta2^{-}$T, $CD4^{+}$ T and NK^{dim} cells in NR and R melanoma patients at different time points of the treatment (n = 8 to 15 per group). P-values were calculated using non-parametric Kruskal-Wallis test (straight lines) or Wilcoxon matched-pairs signed rank test with Bonferroni correction (dashed lines). Only significant statistics are displayed on graphs. (d) Correlation matrix between immune cell proportions in the circulation of NR (upper panel) and R (lower panel) melanoma patients. Black stars illustrate Spearman's correlations with significant P-values after Holm's correction.

Supplementary Figure 3

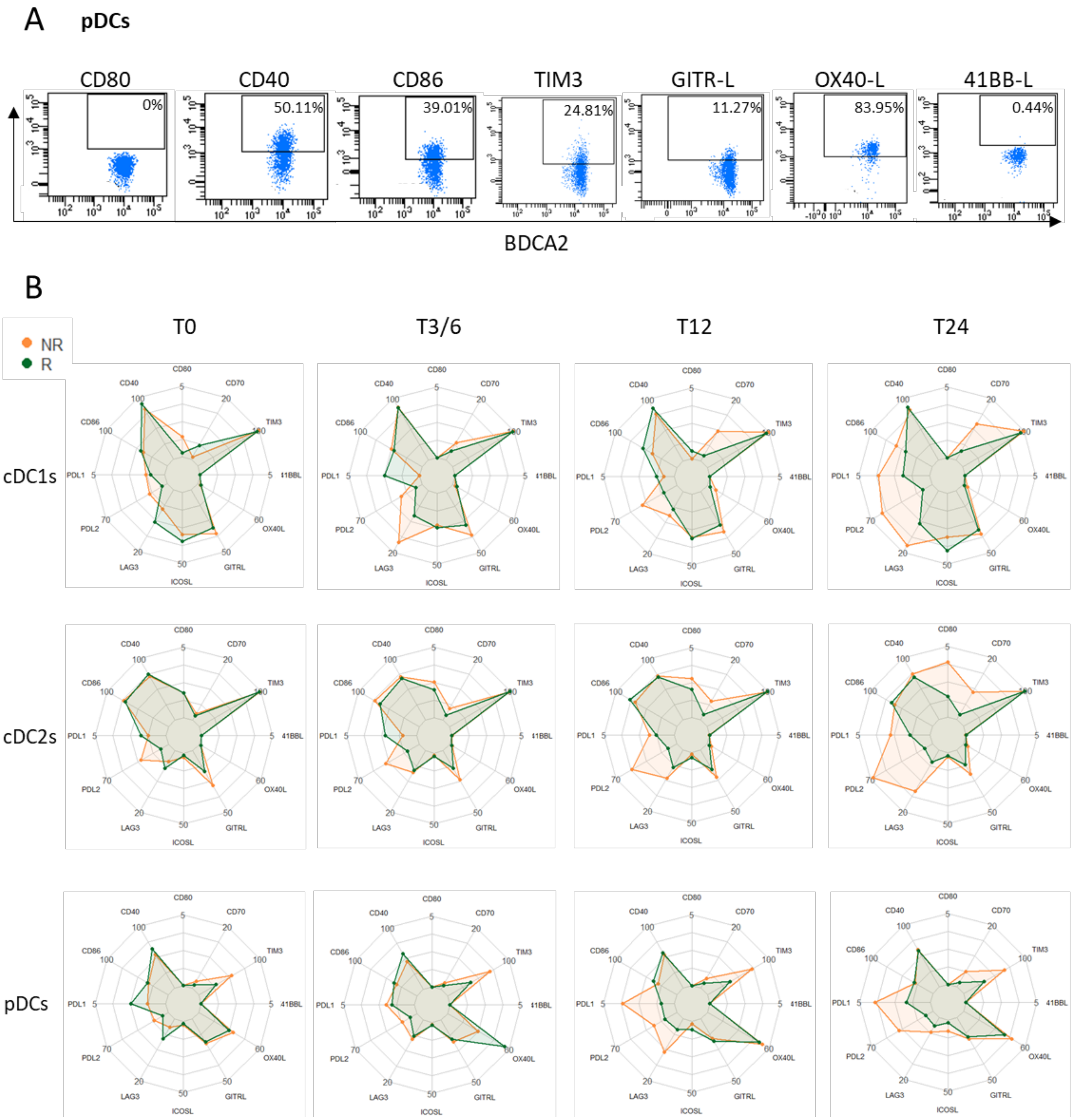

Supplementary Figure 3

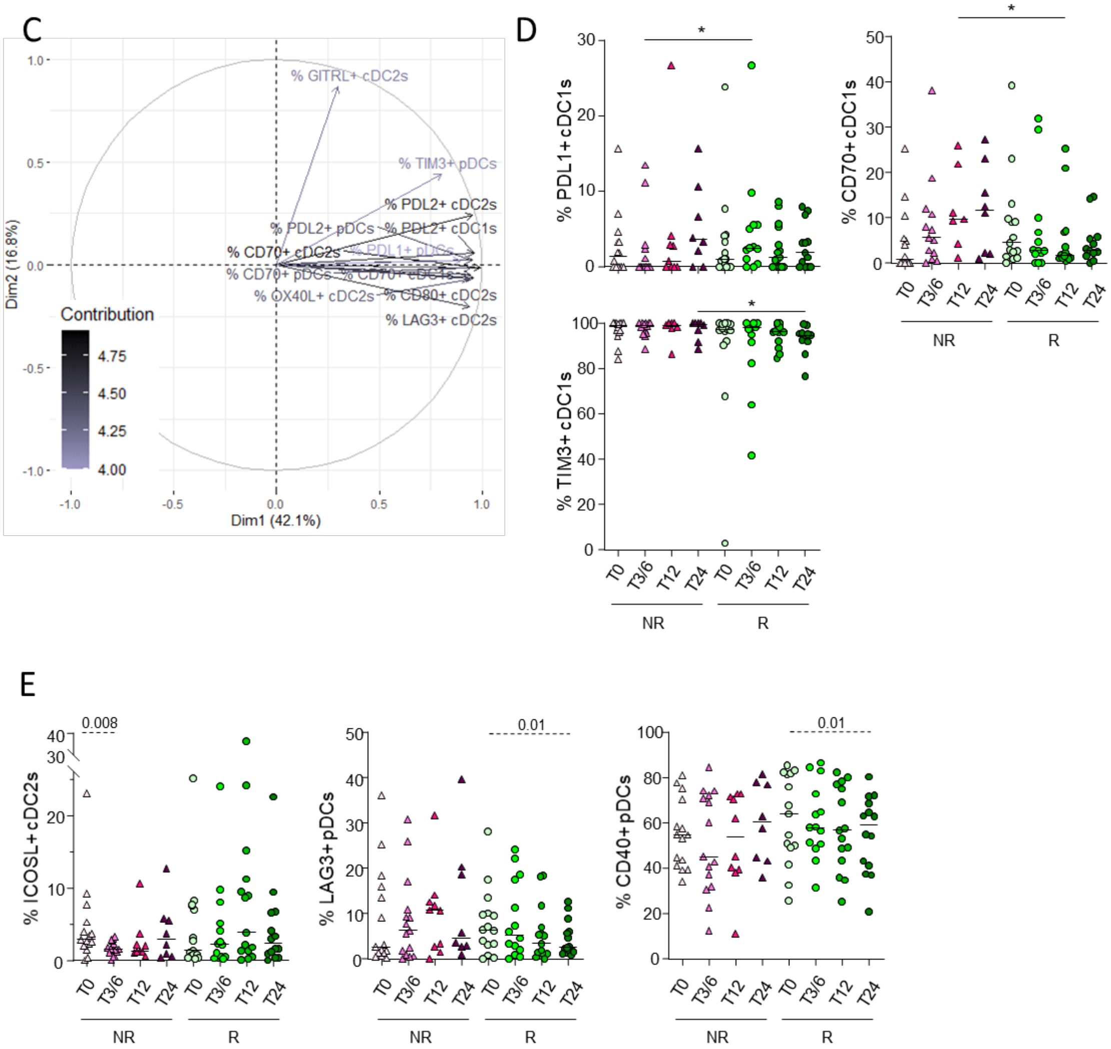

Supplementary Figure 3. DC subsets exhibited distinct expression profiles of ICPs during the course of the treatment according to patients' clinical response to anti-PD1. To study the potential immune specificities of responders to anti-PD1 immunotherapy compared to non-responders, the activation status and ICP expression profile of circulating DC subsets from melanoma patients were assessed using multi-parametric flow cytometry. (a) Representative dot plots displaying the activation status (CD80, CD40 and CD86) and ICP expression (TIM3, GITR-L, OX40-L and 41BB-L) of patients' circulating pDCs. Dot plots pre-gated on alive $CD45^{+}Lin^{-}HLA\text{-}DR^{+}CD11c^{-}BDCA2^{+}$ cells. (b) Radar plots showing the median proportions of DCs expressing the studied activation markers and ICPs (cDC1s [upper panels], cDC2s [middle panels] and pDCs [lower panels]) in non-responder (NR, orange line) and responder (R, green line) patients at different time points of the treatment (T0, T3/6, T12, T24). (c) Graph of variables relative to the PCA analysis shown on Figure 2c. (d) Frequencies of PD-L1^{+}, CD70^{+} or TIM3^{+} cDC1s in NR (triangles) and R (circles) melanoma patients at different time points of the treatment (n = 8 to 15 per group). (e) Frequencies of ICOS-L^{+} cDC2s, and LAG3^{+} or CD40^{+} pDCs in NR (triangles) and R (circles) melanoma patients at different time points of the treatment (n = 8 to 15 per group). (d-e) Bars indicate median. P-values were calculated using non-parametric Kruskal-Wallis test (straight lines) or Wilcoxon matched-pairs signed rank test with Bonferroni correction (dashed lines). Only significant statistics are shown on graphs. *P ≤ 0.05.

Supplementary Figure 4

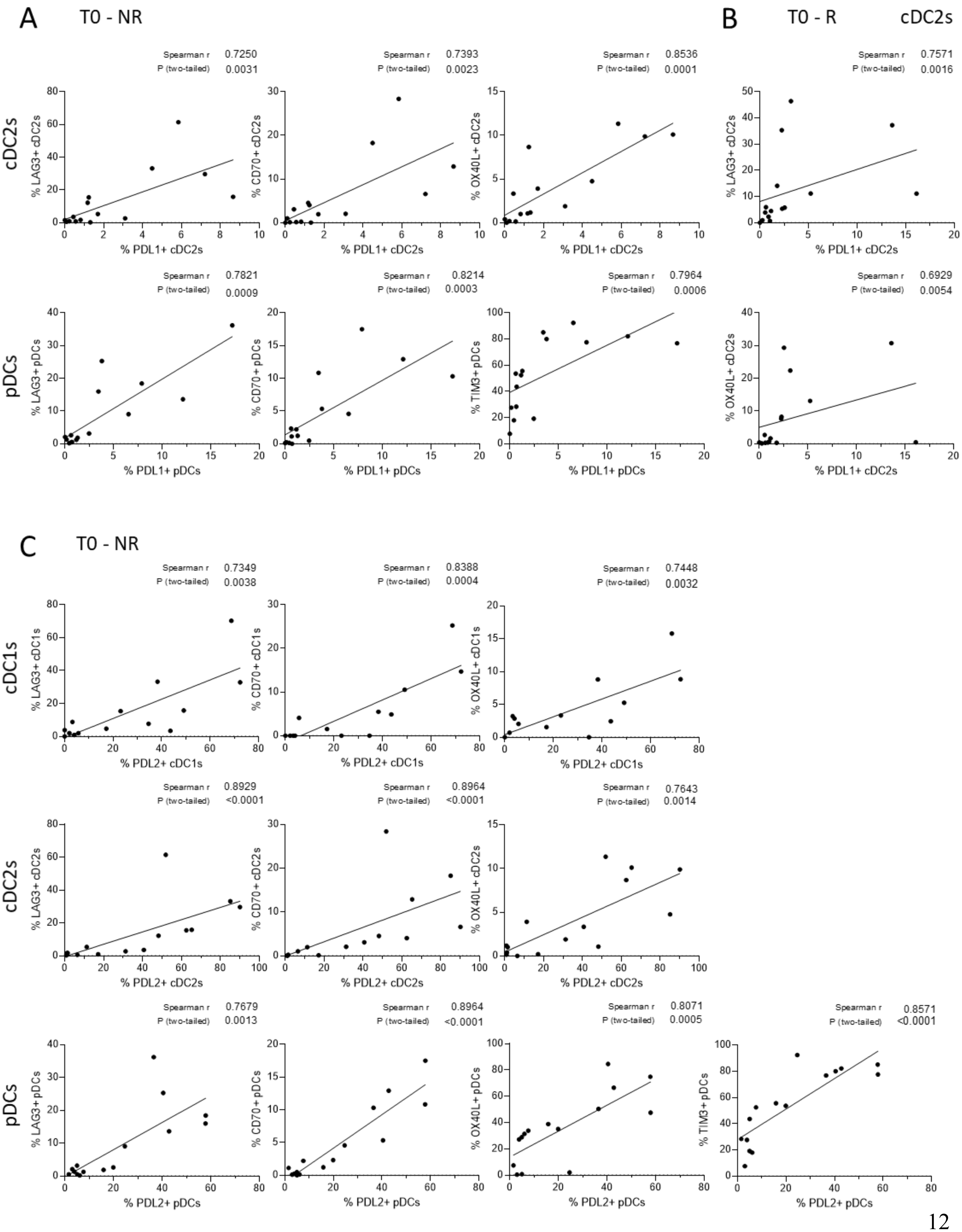

Supplementary Figure 4. Specific correlations between PD-L1/-L2 expression and other ICP profiles by circulating DC subsets of melanoma patients before the start of immunotherapy. Spearman's correlations were performed to assess the link between PD-L1/-L2 expression and other ICPs expressed by each DC subset in non-responder and responder melanoma patients before the start of immunotherapy. (a) Spearman's correlation between the proportions of circulating $LAG3^+$, $CD70^+$ or $OX40\text{-}L^+$ cDC2s (upper panels), or $LAG3^+$, $CD70^+$ or $TIM3^+$ pDCs (lower panels) and the respective frequencies of PD-L1-expressing cDC2s or pDCs in non-responder melanoma patients before immunotherapy (T0-NR; n = 14 to 15). (b) Spearman's correlation between the frequencies of circulating $LAG3^+$ or $OX40\text{-}L^+$ cDC2s and the proportion of $PD\text{-}L1^+$ cDC2s in responder melanoma patients before immunotherapy (T0-R; n = 15). (c) Spearman's correlation between the proportions of circulating $LAG3^+$, $CD70^+$ or $OX40\text{-}L^+$ cDC1s (upper panels), $LAG3^+$, $CD70^+$ or $OX40\text{-}L^+$ cDC2s (middle panels) or $LAG3^+$, $CD70^+$, $OX40\text{-}L^+$ or $TIM3^+$ pDCs (lower panels) and the respective frequencies of PD-L2-expressing cDC1s, cDC2s or pDCs in non-responder melanoma patients before immunotherapy (T0-NR; n = 13 to 15). Significance threshold was set after Bonferroni correction and only significant correlations were illustrated in this figure.

Supplementary Figure 5

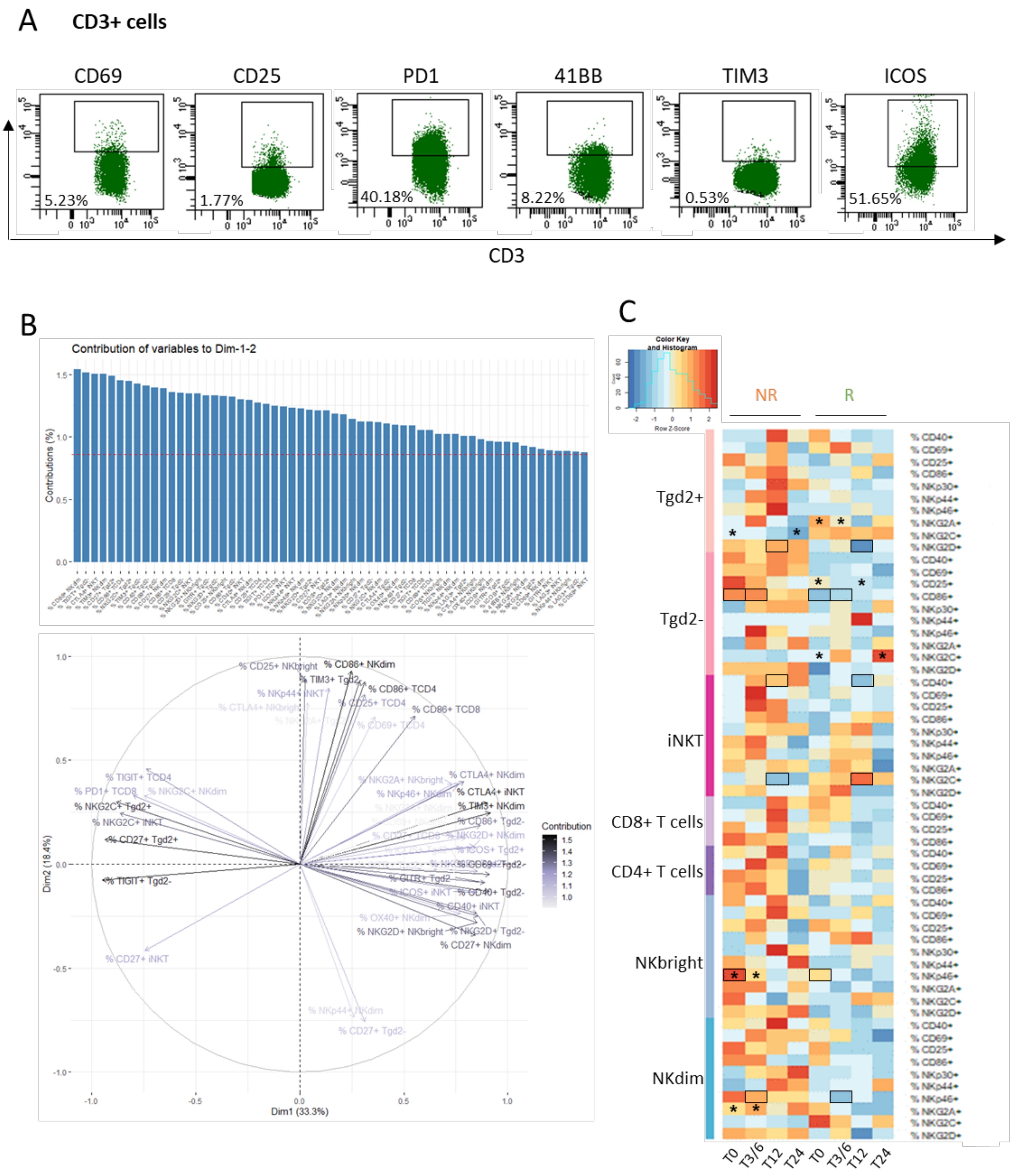

Supplementary Figure 5

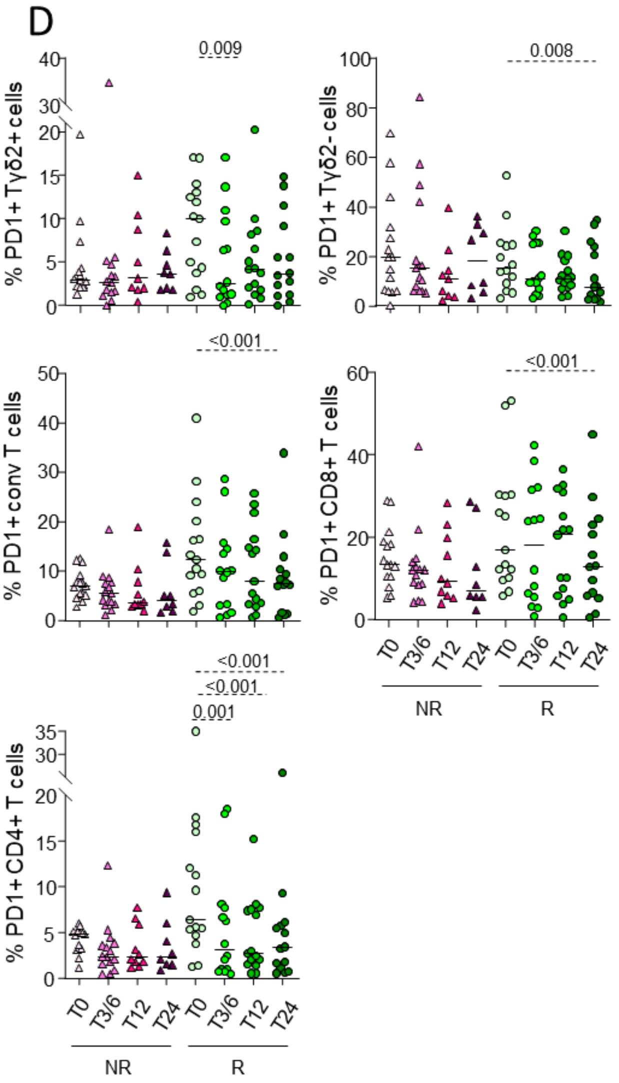

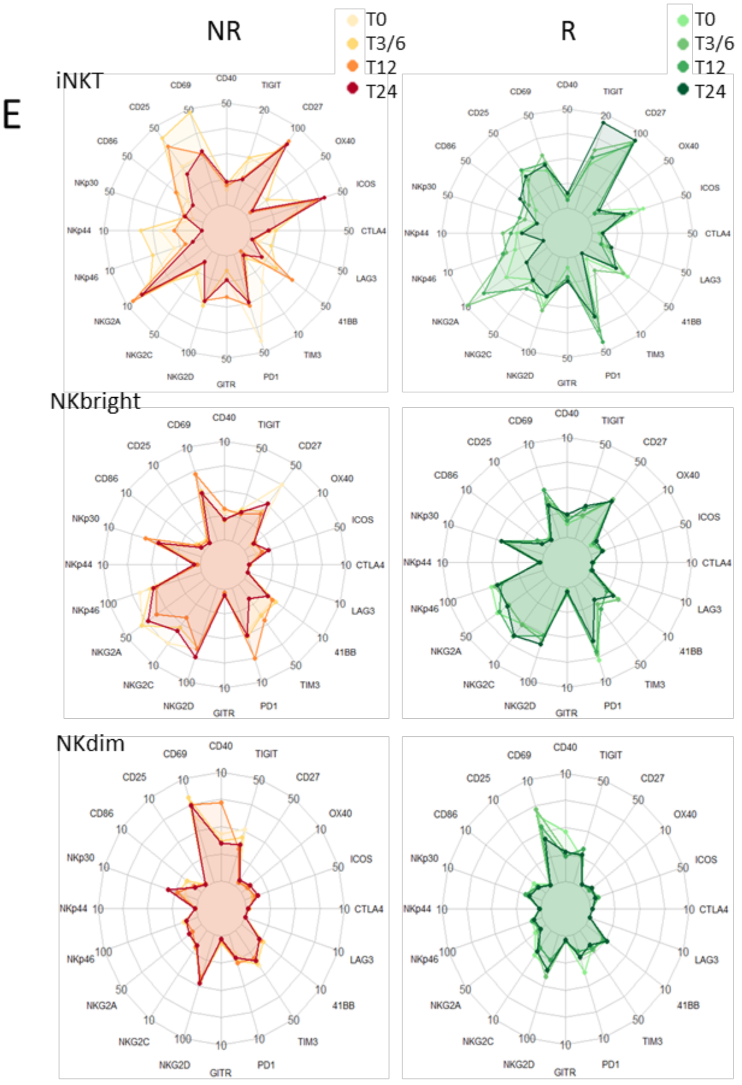

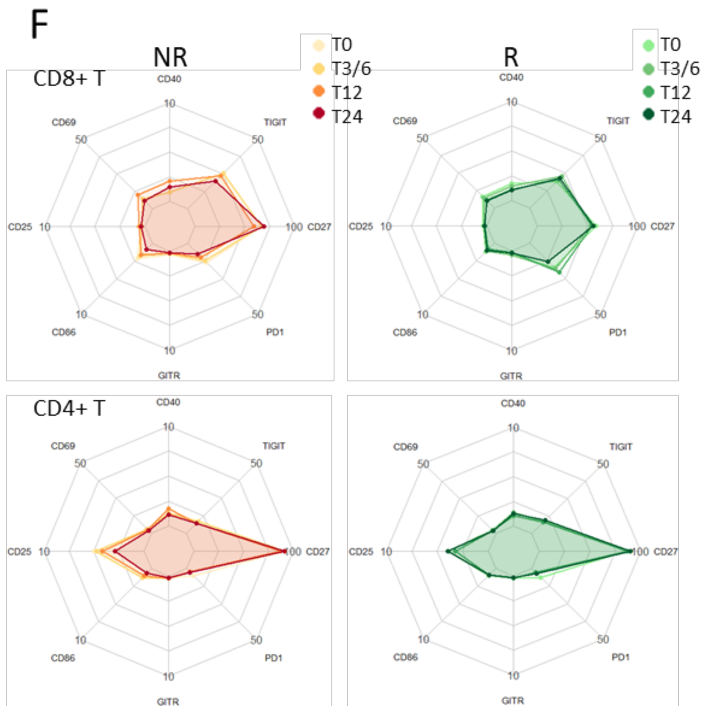

Supplementary Figure 5

G

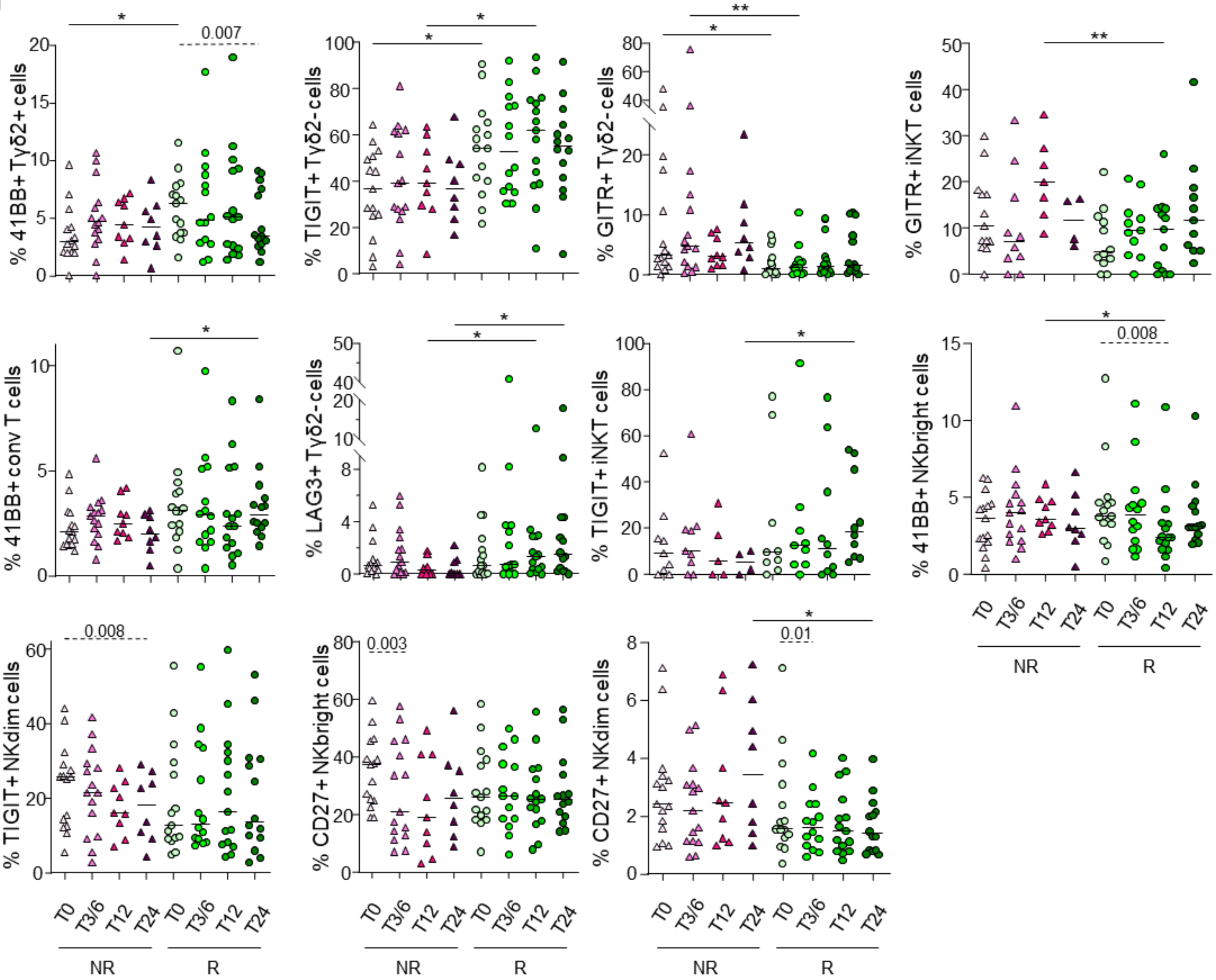

H

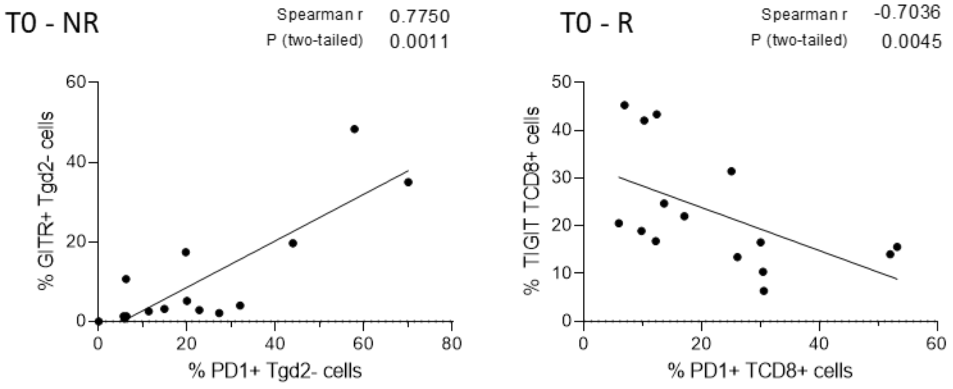

Supplementary Figure 5

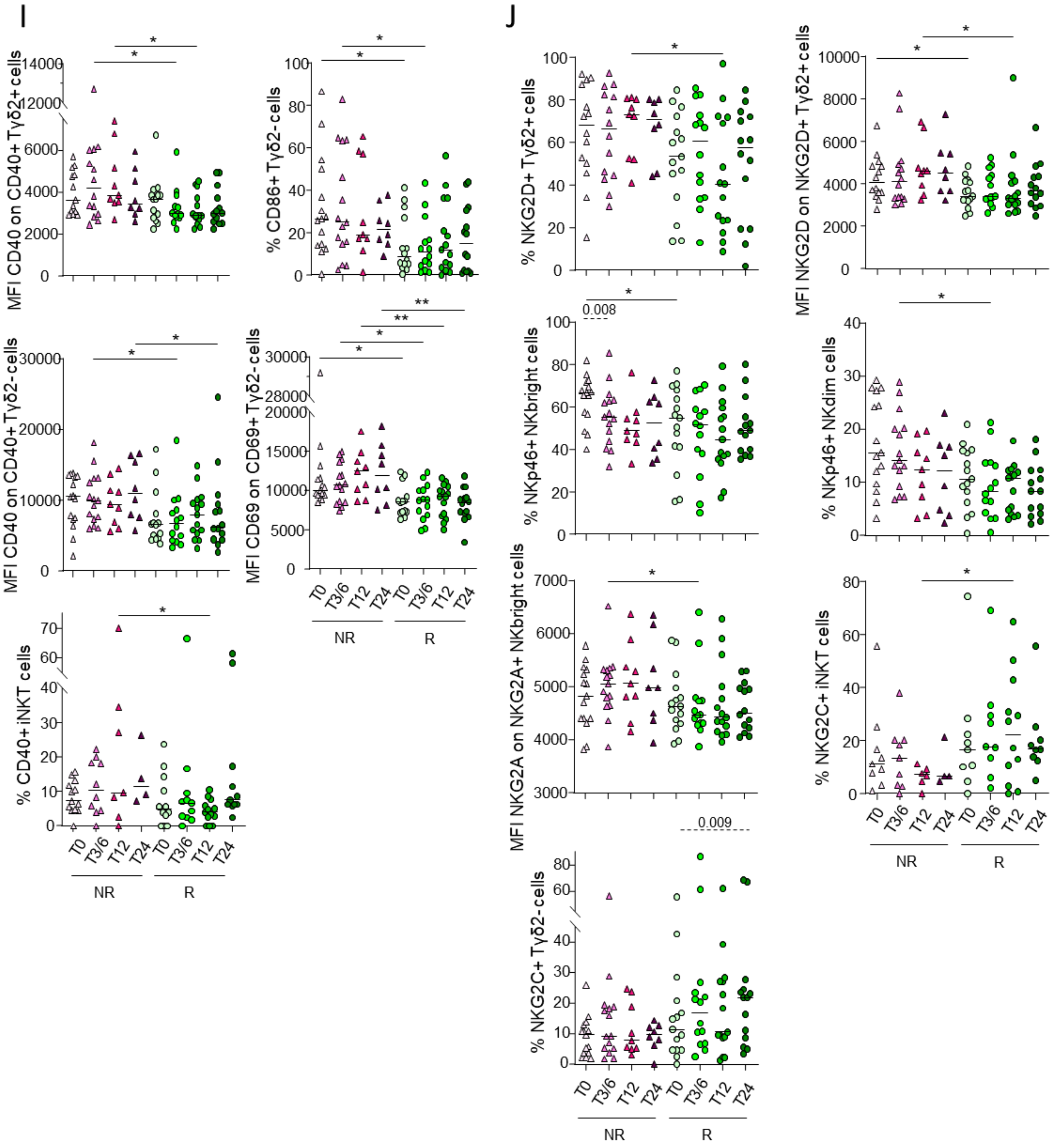

Supplementary Figure 5. Responder and non-responder melanoma patients displayed differences in the activation status, ICP and NCR expression of circulating immune effector cells. To investigate the possible differences between non-responders and responders patients to anti-PD1 therapy, the activation status, ICP and NCR expression profiles of circulating immune effector cells from melanoma patients were investigated using multi-parametric flow cytometry. (a) Representative dot plots displaying the activation status (CD69 and CD25) and ICP expression (PD1, 41BB, TIM3 and ICOS) by circulating $CD3^+$ cells of patients. Dot plots are pre-gated on alive $CD45^+$ cells. (b) Contribution of variables to PC1 and PC2 (upper panel) and graph of variables (lower panel) relative to the PCA analysis shown on Figure 3a. (c) Heat map based on the median frequencies of effector cells expressing activation markers and NCR in patient's blood (NR: non-responders; R: responders) at different time points of the treatment (T0, T3/6, T12, T24). Statistically significant comparisons between patient groups (inter-groups) are showed as black squares (non-parametric Kruskal-Wallis test), and the ones between T0 and another time point in a specific patient group (NR or R patient group, intra-groups) are illustrated as black stars (Wilcoxon matched-pairs signed rank test with Bonferroni correction). (d) Frequencies of PD1-expressing $\gamma\delta2^+$T, $\gamma\delta2^-$T, T_{conv}, $CD8^+$ T and $CD4^+$ T cells in NR (triangles) and R (circles) melanoma patients at different time points of the treatment (n = 8 to 15 per group). (e-f) Radar plots showing the median proportions of effector cells expressing the studied activation markers and ICPs (iNKT, NK^{bright} and NK^{dim}, and $CD8^+$T and $CD4^+$T cells) in NR (left panels) and R (right panels) melanoma patients at different time points of the treatment. (g) Frequencies of $41BB^+$, $TIGIT^+$, $GITR^+$, $LAG3^+$, $CD27^+$ and/or $ICOS^+$ $\gamma\delta2^+$T, $\gamma\delta2^-$T, iNKT, T_{conv}, NK^{bright} and/or NK^{dim} cells in NR (triangles) and R (circles) melanoma patients at different time points of the treatment (n = 4 to 15 per group). (h) Spearman's correlation between the proportions of circulating GITR- and PD1-expressing γδ2-T cells (left panel), and TIGIT- and PD1-expressing $CD8^+$ T cells (right panel) in respectively non-responder or responder melanoma patients before immunotherapy (T0; n = 14 to 15). Significance threshold was set after Bonferroni correction and only significant correlations were illustrated in this figure. (i) Proportions and/or MFI of CD40, CD86 and CD69 on $CD40^+$, $CD86^+$ or $CD69^+$ $\gamma\delta2^+$T, $\gamma\delta2^-$T and/or iNKT cells in NR (triangles) and R (circles) melanoma patients at different time points of the treatment (n = 4 to 15 per group). (j) Frequencies and/or MFI of NKG2D, NKp46, NKG2A or NKG2C on NKG2D-, NKp46-, NKG2A- or NKG2C-expressing $\gamma\delta2^+$T, NK^{bright}, NK^{dim}, iNKT and/or $\gamma\delta2^-$T cells in NR (triangles) and R (circles) melanoma patients at different time points of the treatment (n = 4 to 15 per group). (d;g;i-j) Bars indicate median. P-values were calculated using non-parametric Kruskal-Wallis test (straight lines) or Wilcoxon matched-pairs signed rank test with Bonferroni correction (dashed lines). Only significant statistics are displayed on graphs. $^*P \leq 0.05$, $^{**}P \leq 0.01$.

Supplementary Figure 6

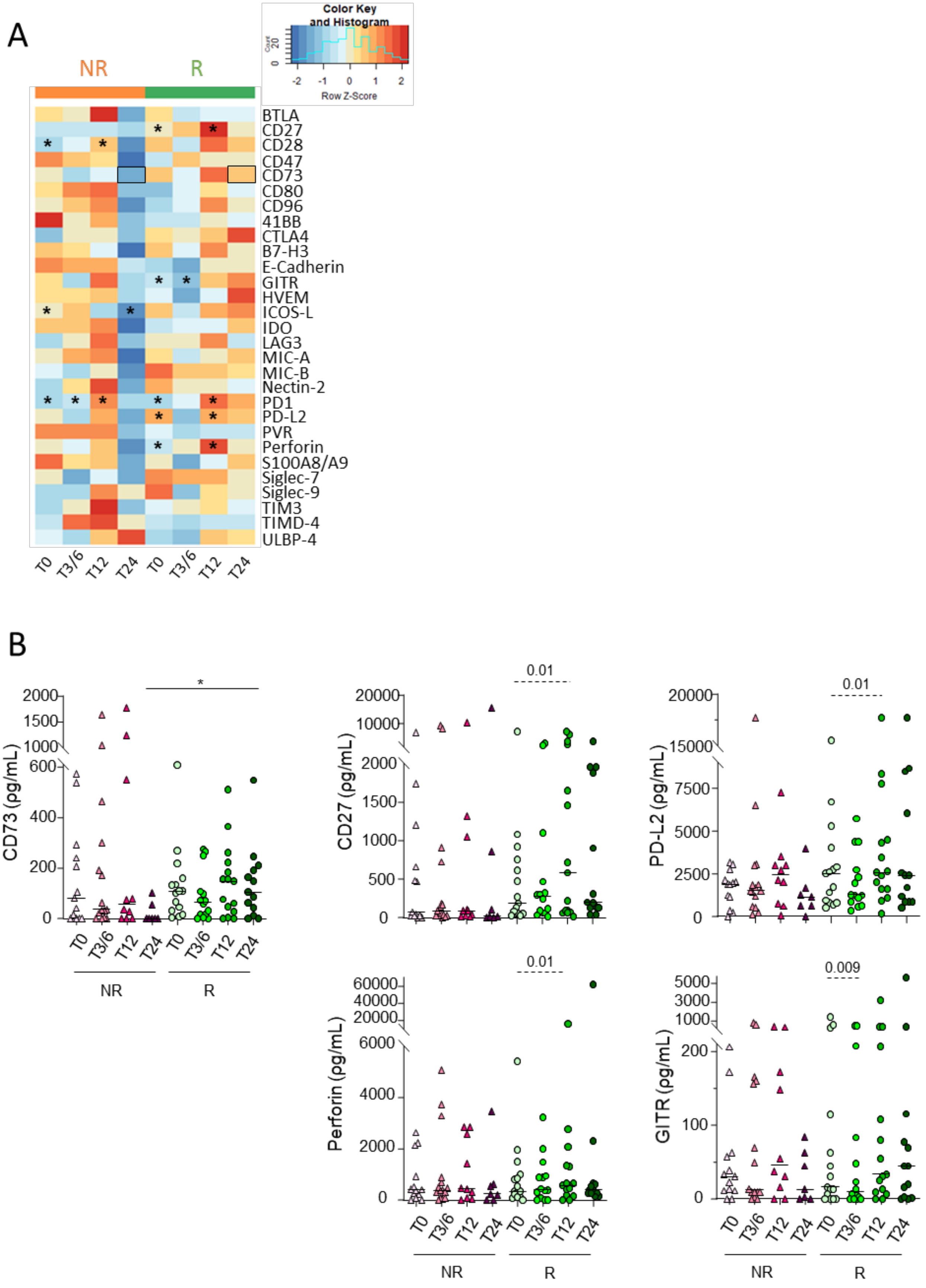

Supplementary Figure 6. Soluble factors found in the plasma of melanoma patients do not allow the distinction of responders from non-responders before the beginning of the treatment. To evaluate the profile of soluble factors (especially soluble immune checkpoints) in the plasma of patients, 37 soluble factors were measured in plasmas before and during the course of anti-PD1 treatment by Luminex technology. (a) Heat map based on the median levels of soluble factors found in the plasma of melanoma patients undergoing immunotherapy (NR: non-responders; R: responders) at different time points of the treatment (T0, T3/6, T12, T24). Statistically significant comparisons between patient groups (inter-groups) are showed as black squares (non-parametric Kruskal-Wallis test), and the ones between T0 and another time point in a specific patient group (NR or R patient group, intra-groups) are illustrated as black stars (Wilcoxon matched-pairs signed rank test with Bonferroni correction). (b) Comparative amounts of CD73, CD27, PD-L2, GITR and perforin from plasma derived from NR and R melanoma patients at different time points of the treatment ($n = 8$ to 15 per group). Bars indicate median. P-values were calculated using non-parametric Kruskal-Wallis test (straight lines) or Wilcoxon matched-pairs signed rank test with Bonferroni correction (dashed lines). Only significant statistics are displayed on graphs. $^*P \leq 0.05$.

Supplementary Figure 7

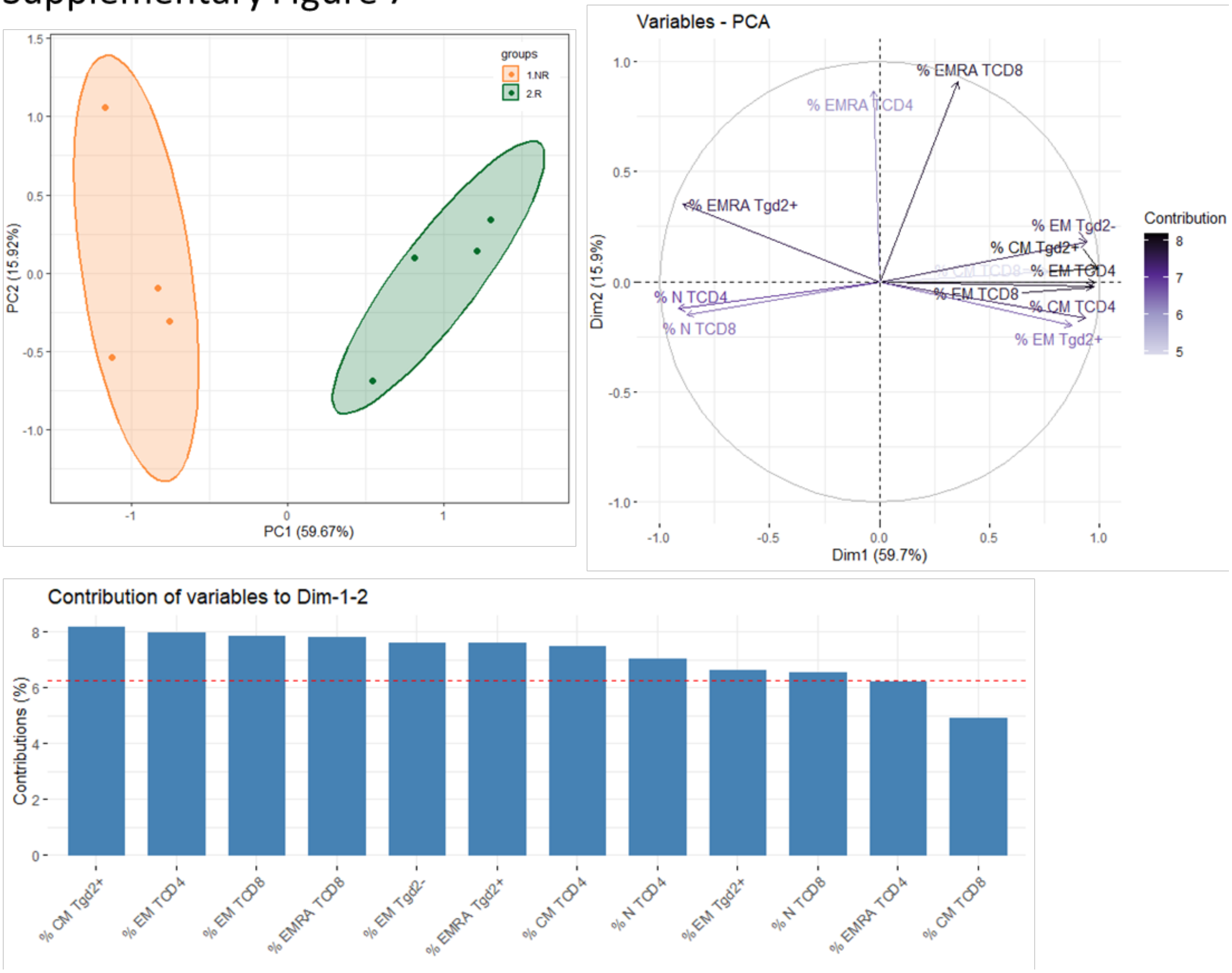

Supplementary Figure 7. The differentiation stage of circulating conventional and γδ T cells distinguished responders and non-responders melanoma patients to immunotherapy. To examine whether T-cell differentiation stage dictated patient's clinical response to anti-PD1, frequencies of N, CM, EM and EMRA populations of $\gamma\delta2^+$T, $\gamma\delta2^-$T, CD8$^+$ T and CD4$^+$ T cells were assessed in melanoma patients undergoing treatment. PCA (upper left panel), graph of variables (upper right panel) and contribution of variables to PC1 and PC2 (bottom panel) relative to the PCA analysis based on the median frequencies of N, CM, EM and EMRA populations of $\gamma\delta2^+$T, $\gamma\delta2^-$T, CD8$^+$ T and CD4$^+$ T cells in patient's blood (NR: non-responders; R: responders) at different time points of the treatment (T0, T3/6, T12, T24).

Supplementary Figure 8

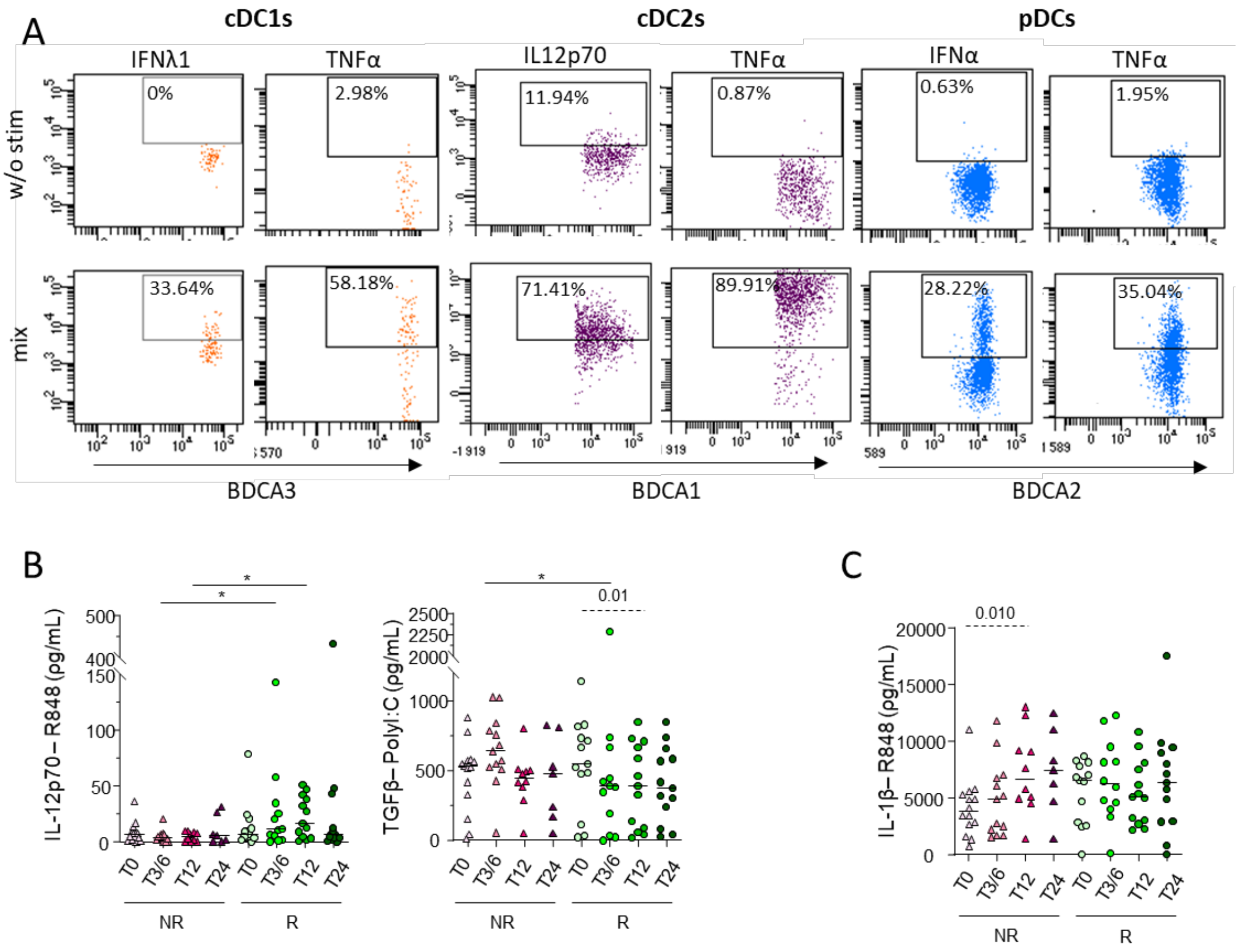

Supplementary Figure 8. Higher levels of IL-12p70 and lower levels of TGF-β1 after TLR stimulation were found in PBMC supernatants derived from melanoma patients responding to immunotherapy when compared to non-responders. To investigate the functionality of DC subsets in melanoma patients following immunotherapy, cytokine/chemokine production and secretion by DCs after TLR stimulation were assessed respectively by intracellular cytokine staining using multi-parametric flow cytometry and ProcartaPlex dosages of culture supernatants using Luminex technology. (a) Representative dot plots displaying the cytokine production (IFN-λ1, IL-12p70, IFN-α and TNF-α) by circulating cDC1s, cDC2s and pDCs derived from melanoma patients upon 5h culture without (upper panels) or with (bottom panels) a mixture of TLR-L (mix; combination of PolyI:C, R848 and CpGA). (b) Levels of IL12p70 and TGF-β1 in culture supernatants after stimulation with single TLR-L (R848, PolyI:C) of PBMC derived from non-responder (NR; triangles) and responder (R; circles) melanoma patients at different time points of the treatment (T0, T3/6, T12, T24; n = 7 to 13 per group). (c) Levels of IL-1β in culture supernatants after stimulation with single TLR-L (R848) or mixture (mix: PolyI:C, R848 and CpGA) of PBMC derived from non-responder (NR; triangles) and responder (R; circles) melanoma patients at different time points of the treatment (T0, T3/6, T12, T24; n = 7 to 13 per group). (b-c) P-values were calculated using non-parametric Kruskal-Wallis test (straight lines) or Wilcoxon matched-pairs signed rank test with Bonferroni correction (dashed lines). Only significant statistics are displayed on graphs. *P ≤ 0.05.

Supplementary Figure 9

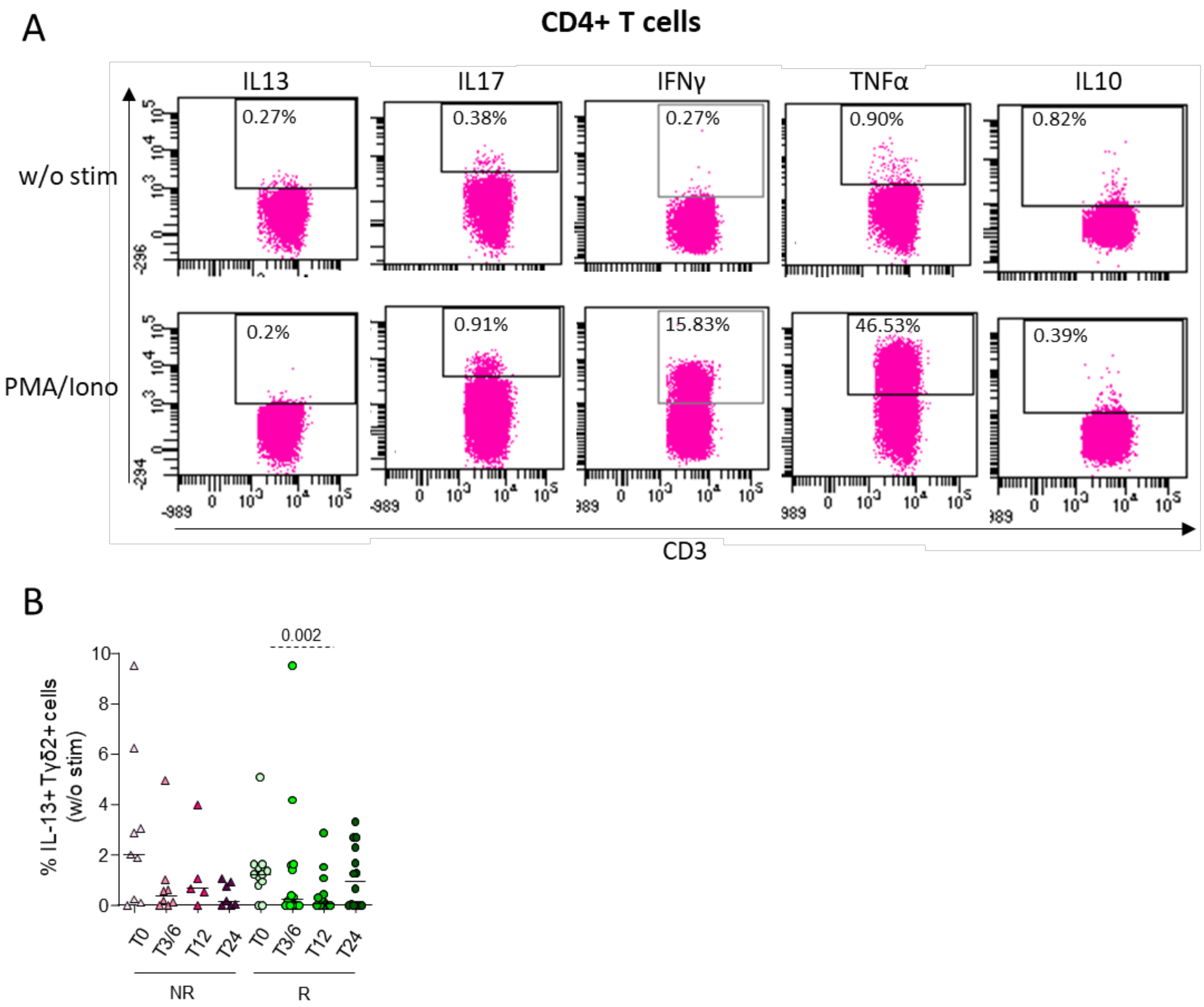

Supplementary Figure 9. Decreased frequencies of IL-13- and TNF-α-producing γδT cells in responder melanoma patients during the course of the treatment. To inspect the functionality of circulating immune effector cells in melanoma patients following immunotherapy, cytokine production by specific cell subsets was assessed by intracellular cytokine staining and multi-parametric flow cytometry after PBMC stimulation (by PMA/Iono, HMB-PP, IL-12/IL-18 or αGalCer) . (a) Representative dot plots displaying the cytokine production (IL-13, IL-17A, IFN-γ, TNF-α and IL-10) by circulating $CD4^+$ T cells derived from melanoma patients following culture of PBMC without (upper panels) or with PMA/Iono (bottom panels). (b) Frequencies of $IL\text{-}13^+$ $γδ2^+$T cells, after culture without any stimulation (w/o stim), derived from NR (triangles) and R (circles) melanoma patients at different time points of the treatment (n = 5 to 14 per group). Bars indicate median. P-values were calculated using non-parametric Kruskal-Wallis test (straight lines) or Wilcoxon matched-pairs signed rank test with Bonferroni correction (dashed lines). Only significant statistics are displayed on graphs.

Supplementary Figure 10

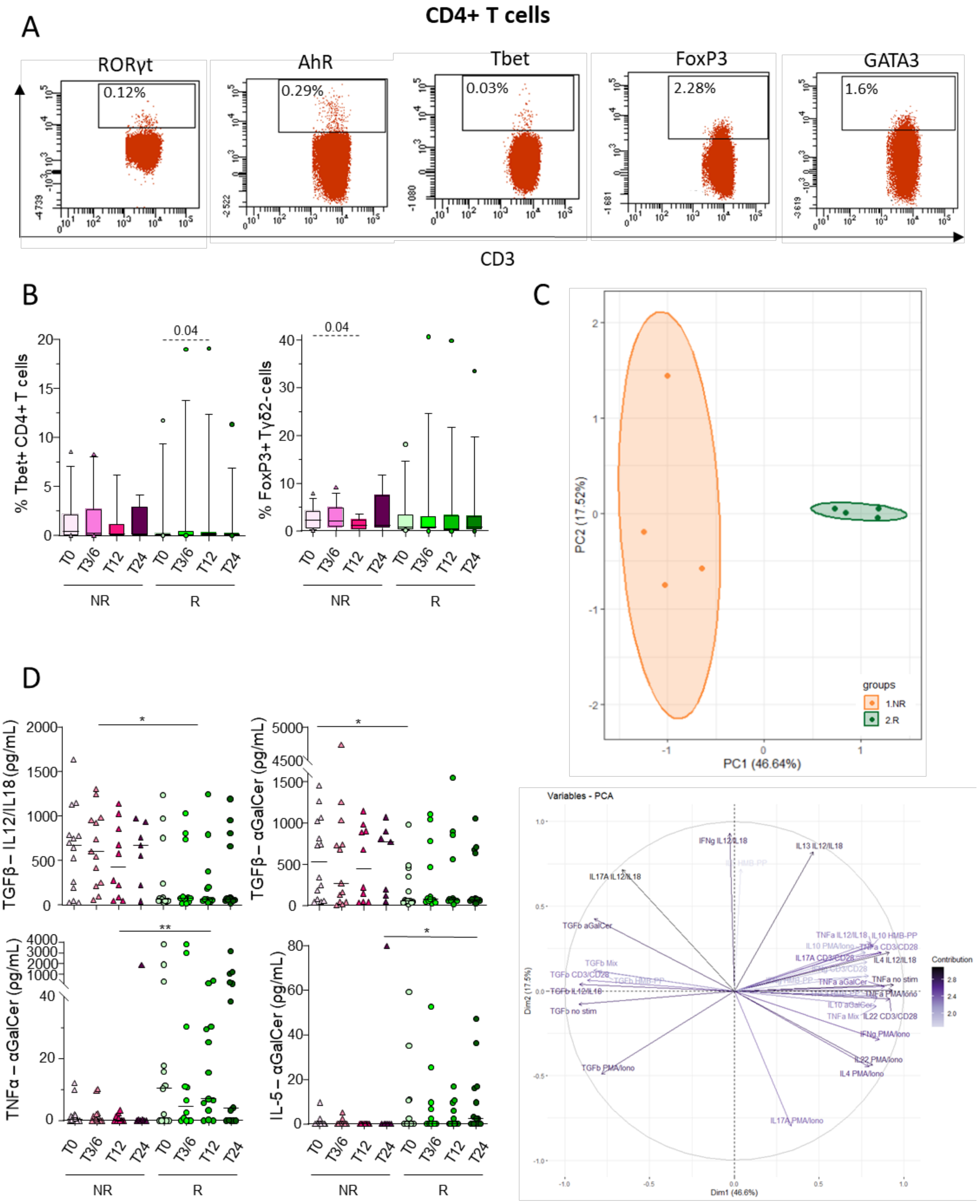

Supplementary Figure 10. Distinct Th orientations of conventional and γδT cells, and specific cytokine secretion upon effector-specific stimulation in melanoma patients responding to anti-PD1 therapy when compared to non-responders. To investigate whether Th profile of conventional and γδT cells differed depending on the patient's response to immunotherapy, frequencies of RORγt, AhR, Tbet, FoxP3 or GATA3-positive $\gamma\delta2^{+}$T, $\gamma\delta2^{-}$T, $CD8^{+}$ T and $CD4^{+}$ T cells were assessed in melanoma patients before and during the course of the treatment following intra-nuclear staining and flow cytometry analysis. Furthermore, to decipher the combined functionality of circulating immune effector cells in melanoma patients following anti-PD1 treatment, cytokine secretion was analyzed in supernatants derived from patient's PBMC after stimulation (by PMA/Iono, alone or combined CD3/CD28, HMB-PP, IL-12/IL-18 and αGalCer) using Luminex technology. (a) Representative dot plots displaying transcription factor expression by circulating $CD4^{+}$ T cells derived from melanoma patients. Dot plots are pre-gated on alive $CD45^{+}CD3^{+}CD4^{+}$ cells. (b) Box and whiskers plots illustrating the frequencies of Tbet-expressing $CD4^{+}$ T cells, and the proportions of $FoxP3^{+}$ $\gamma\delta2^{-}$T cells derived from non-responder (NR; triangles) and responder (R; circles) melanoma patients at different time points of the treatment (T0, T3/6, T12, T24; n = 8 to 15 per group). (c) PCA (upper panel) and graph of variables (lower panel) relative to the PCA analysis based on the median of the levels of cytokines found in supernatants after culture of PBMC with different stimulants and derived from patient's blood (NR: non-responders; R: responders) at different time points of the treatment (T0, T3/6, T12, T24). (d) Levels of TGF-β1 found after culture with different stimulants (IL-12/IL-18 or αGalCer), and levels of TNF-α and IL-5 found after culture with αGalCer of PBMC derived from NR (triangles) and R (circles) melanoma patients at different time points of the treatment (n = 7 to 14 per group). (b;d) P-values were calculated using non-parametric Kruskal-Wallis test (straight lines) or Wilcoxon matched-pairs signed rank test (dashed lines). Only significant statistics are displayed on graphs. $^{*}P \leq 0.05$, $^{**}P \leq 0.01$.

Supplementary Figure 11

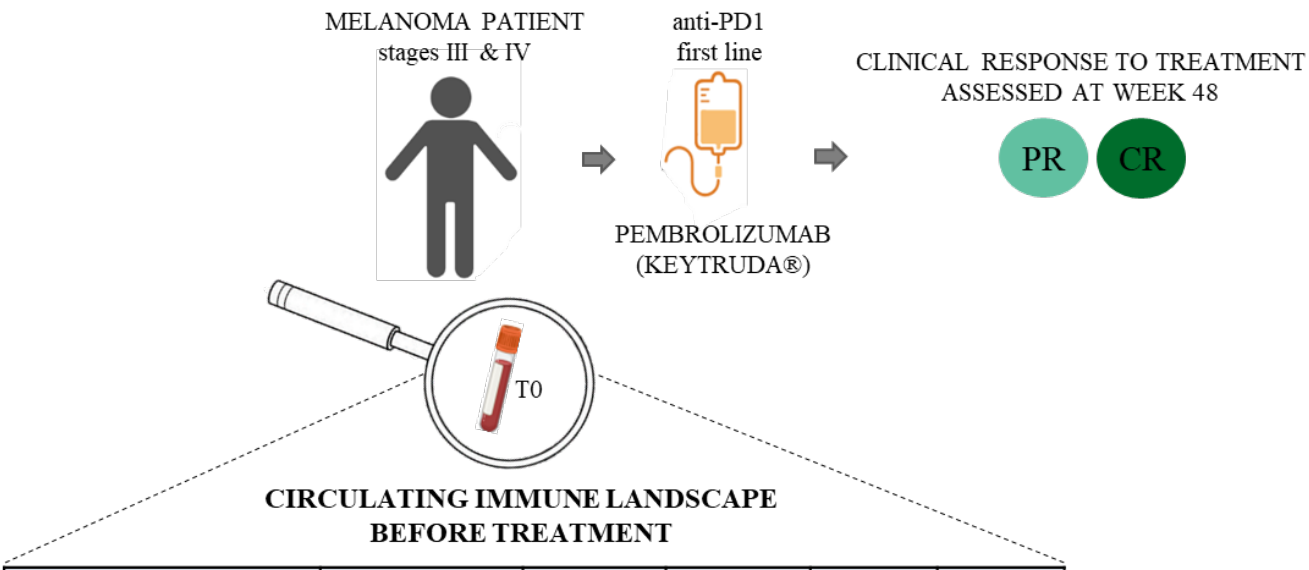

Cell subsets	Parameters within cell subsets	Level	Median	Min	Max
**CD45+ cells**	% cDC1s	High	0.033	0.002	0.060
**CD4+ T cells**	% PD1+	High	6.379	1.340	34.88
**Tγδ2+ cells**	% 41BB+	High	6.295	1.603	11.51
	% NKG2D+	Low	53.39	13.53	84.88
	% CM	High	13.60	0	53.67
	% EMRA	Low	13.16	4.491	40.91
**Tγδ2- cells**	% TIGIT+	High	54.09	21.39	90.49
	% CD69+	Low	18.46	4.416	72.61
	% CD86+	Low	8.678	0.512	41.18
	% GITR+	Low	1.076	0	6.626
**NK CD56^{bright} cells**	% NKp46+	Low	54.75	15.39	77.08

Supplementary Figure 11. Key differences in the immune profile of responder melanoma patients before treatment

2.2 Supplementary Tables

Supplementary Table 1. Characteristics of melanoma patients

**Characteristic**	**Melanoma (*n* = 30)**
Age	
mean, median (range)	72, 74 (36-93)
Sex, *n* (%)	
Male	18 (60)
Female	12 (40)
Breslow, *n* (%)	
< 1 mm	2 (6.66)
1 - 2 mm	3 (10)
2 – 4 mm	8 (26.67)
> 4 mm	8 (26.67)
unknown	9 (30)
Clark, *n* (%)	
II	1 (3.33)
III	2 (6.66)
IV	7 (23.33)
V	4 (13.33)
unknown	16 (53.33)
Ulceration, *n* (%)	
Yes	10 (33.33)
No	5 (16.67)
unknown	15 (50)
Metastasis stage, *n* (%)	
M1a	6 (20)
M1b	7 (23.33)
M1c	6 (20)
M1d	10 (33.33)
unknown	1 (3.33)
Previous treatments, *n* (%)	
none	3 (10)
surgery	27 (90)
radiotherapy	7 (23.33)
LDH levels measured before anti-PD1 therapy, n (%)	
higher than normal LDH levels	11 (36.67)
normal LDH levels	19 (63.33)
Response to anti-PD1 therapy measured at 48weeks, *n* (%)	
PD	14 (46.67)
SD	1 (3.33)
PR	9 (30)
CR	6 (20)

Abbreviations: CR, complete response; PD, progressive disease; PR, partial response; SD, stable disease
